# Orphan Crops Browser: a bridge between model and orphan crops

**DOI:** 10.1007/s11032-015-0430-2

**Published:** 2016-01-12

**Authors:** Claire Lessa Alvim Kamei, Edouard I. Severing, Annemarie Dechesne, Heleen Furrer, Oene Dolstra, Luisa M. Trindade

**Affiliations:** Wageningen UR Plant Breeding, Wageningen University and Research Centre, Droevendaalsesteeg 1, 6708 PB Wageningen, The Netherlands; Laboratory of Genetics, Wageningen University and Research Centre, Droevendaalsesteeg 1, 6708 PB Wageningen, The Netherlands; Department of Comparative Development and Genetics, Max Planck Institute for Plant Breeding Research, Carl-von-Linné-Weg 10, 50829 Cologne, Germany; Department of Plant Developmental Biology, Max Planck Institute for Plant Breeding Research, Carl-von-Linné-Weg 10, 50829 Cologne, Germany

**Keywords:** Orthologous genes, Bioinformatics tool, Breeding targets, De novo transcriptome, Orphan crops

## Abstract

Many important crops have received little attention by the scientific community, either because they are not considered economically important or due to their large and complex genomes. De novo transcriptome assembly, using next-generation sequencing data, is an attractive option for the study of these orphan crops. In spite of the large amount of sequencing data that can be generated, there is currently a lack of tools which can effectively help molecular breeders and biologists to mine this type of information. Our goal was to develop a tool that enables molecular breeders, without extensive bioinformatics knowledge, to efficiently study de novo transcriptome data from any orphan crop (http://www.bioinformatics.nl/denovobrowser/db/species/index). The Orphan Crops Browser has been designed to facilitate the following tasks (1) search and identification of candidate transcripts based on phylogenetic relationships between orthologous sequence data from a set of related species and (2) design specific and degenerate primers for expression studies in the orphan crop of interest. To demonstrate the usability and reliability of the browser, it was used to identify the putative orthologues of 17 known lignin biosynthetic genes from maize and sugarcane in the orphan crop *Miscanthus sinensis*. Expression studies in miscanthus stem internode tissue differing in maturation were subsequently carried out, to follow the expression of these genes during lignification. Our results showed a negative correlation between lignin content and gene expression. The present data are in agreement with recent findings in maize and other crops, and it is further discussed in this paper.

**Electronic supplementary material:**

The online version of this article (doi:10.1007/s11032-015-0430-2) contains supplementary material, which is available to authorized users.

## Introduction

In developing countries, several indigenous plant species form the basis of subsistence to many local and regional communities, providing food, animal feed and other non-food products. These crops are well accepted and preferred by farmers and consumers and well adapted to the local conditions. However, their cultural and agricultural importance is undervalued. Given their lack of representation on the global markets, the research investment by public and private sectors is just a very small fraction of what is invested in major arable crops, such as maize, rice and wheat, more economically important to Europe and the USA (Jonkers 2010). In addition, the commonly large, complex and polyploidy genomes of these “orphan crops” also discourage further research. Given these challenges, one alternative to progress in orphan crop research would be to make use of knowledge available from model species, translating findings from models to orphans. To facilitate this task, breeding and biotechnological tools to study complex genomes and the transcriptomes of many orphan crops are currently being developed. Transcriptome datasets are valuable sources of information and useful to unravel plant pathways during different developmental stages and in response to biotic or abiotic stresses. The use of transcriptome datasets from closely related species will allow deepening our current knowledge on processes fundamental to the development of better and more competitive crops. For example, the orphan crop miscanthus is a new and promising C4 grass suitable for the production of second-generation biofuels. The crop comprises different polyploid species, including *Miscanthus sinensis* and *M. x giganteus*, and still lacks a reference assembled genome. Several researchers, however, have recently explored their transcriptional profile as an alternative to whole-genome sequencing (Barling et al. 2013; Chouvarine et al. 2012; Kim et al. 2014; Straub et al. 2013a; Swaminathan et al. 2012).

Commonly, transcriptome profiling consists of several steps: first, transcript fragments are sequenced using next-generation sequencing technologies such as Illumina and 454. Second, the transcriptome is reconstructed by performing *de novo* assemblies using dedicated software such as Trinity (Grabherr et al. 2011). Third, the de novo transcriptome is annotated using tools such as Blast2GO (Conesa et al. 2005). These steps are followed by the search of interesting genes and confirmation steps using wet-lab approaches. To this end, the knowledge gained from other organisms is often used to identify genes of interest in their *de novo* transcriptome. There are tools that provide user-friendly interfaces for gene discovery in de novo transcriptomes such as Trapid (Van Bel et al. 2013) and Trinotate-web (http://trinotate.github.io/TrinotateWeb.html). Although these tools are very powerful in helping to explore *de novo* transcriptome, they stop at the point where the user has a potential target gene and they do not provide the means of producing primers to confirm their presence.

Here we present the Orphan Crop Browser: a novel molecular breeding tool specifically designed to search for genes in species where little genome sequencing information is available using data available from other species (http://www.bioinformatics.nl/denovobrowser/db/species/index). To demonstrate the applicability of this newly developed tool, we focused on the search of miscanthus orthologues from maize and sugarcane genes known to operate in the lignin biosynthetic pathway.

## Results

A browser to facilitate the study and to assist molecular breeding of orphan crops is presented. This tool enables to quickly identify target genes in their de novo transcriptome through a user-friendly graphical interface. In addition, the browser aids in the design and testing of primers that can be used for confirming the existence and quantifying the abundance of the target genes molecularly. This feature is included in the browser to avoid unnecessary copying and pasting between programs and Web sites. The result section is structured as follows: First, we describe the information that can be used as input for the browser; secondly, an overview of the various functionalities of the browser is given, and finally the usefulness and potentialities of this browser are illustrated using a biological example focusing on the orphan crop *Miscanthus sinensis*.

### Browser setup

Before the browser can be used, a database has to be constructed that contains all information available from the novel-(orphan crop) and several annotated species. A graphical interface has specifically been developed to simplify the creation of the database. Different types of data can be uploaded into the browser including sequences, annotations, gene function information and homologous sequence clusters. A brief description of the input data is provided below.

Sequence data are the most basic type of information required before the browser can be used. The browser has been designed to handle full-length mRNA, coding and protein sequences. Users are required to provide both coding sequences and the corresponding protein translations for each species to the browser, since orthologous inferences in the browser are performed using predicted proteins and their corresponding coding sequences. Full-length mRNAs have no effect on the browser performance because they are not directly used for orthology inference. As a result, their inclusion in the browser is optional. The browser also includes the possibility to integrate exon/intron structure information. Information about the gene structure can help users to detect crop-specific alternative splicing events.

Different types of annotations can be uploaded into the browser. Although all sequences that are uploaded into the database receive an internal name, their original identifier is preserved as part of its annotation. Function descriptions and Gene Ontology term annotations, obtained using programs such as Blast2GO, enable users to search the database for specific key words. Users can upload tables containing the sequence IDs, descriptions and gene ontology (GO) (Ashburner et al. 2000) terms into the database. The most advanced type of annotation that can be uploaded into the database is protein domain annotations such as derived from PFAM (Punta et al. 2012). Protein domains correspond to conserved regions of proteins for which several have a known function, and they can be searched on both their ID or description.

Predefined clusters of homologous sequence can also be uploaded into the database, representing groups of orthologous proteins or gene families. Construction of these clusters can be done using programs such as OrthoMCL (Li et al. 2003). Although the inclusion of predefined sequence clusters enables users to quickly find related sequences, they are not strictly necessary for efficient use of the browser.

### Functionalities

**Fig. 1:**
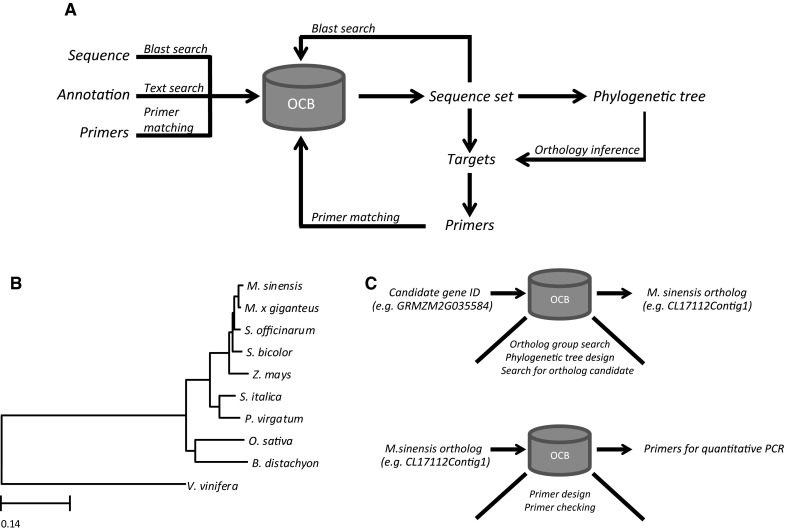
Schematic work flow and use of the Orphan Crop Browser. **a** Main work flow of the Orphan Crops Browser. Using blast-, text- or primer-search sequences are identified in the browser. The initial search results can subsequently be used to create a set of related sequences, which can be used for constructing phylogenetic trees. Target genes can be identified by visually inferring orthologous relationships in the trees. After identification of targets, primers can be designed and tested for uniqueness by matching them against the database sequences. **b** Maximum likelihood tree of monocot species used in this study, including *Vitis vinifera* as an out-group. **c** Schematic representation of adopted strategy to identify miscanthus orthologous genes with the aid of the Orphan Crops Browser (OCB) and design of primers for qPCR

The functionalities of the browser are based on a specific work flow (Fig. 1a) which can be subdivided into five phases: (1) Initial search: users will search sequences within the database using the text or similarity search modules; (2) Creation of a set of related sequences: a set of related sequences is constructed for phylogenetic analysis; (3) Phylogenetic analysis: here users generate multiple sequence alignments and construct phylogenetic trees for identifying the target sequences; (4) Primer-design phase: primers are designed for wet-lab confirmation of sequences; (5) Primer testing phase: in this last phase, users test whether the primer pairs are adequate for finding the intended targets. Here, we provide a brief description of the different modules of the browser.

### Text search

**Fig. 2:**
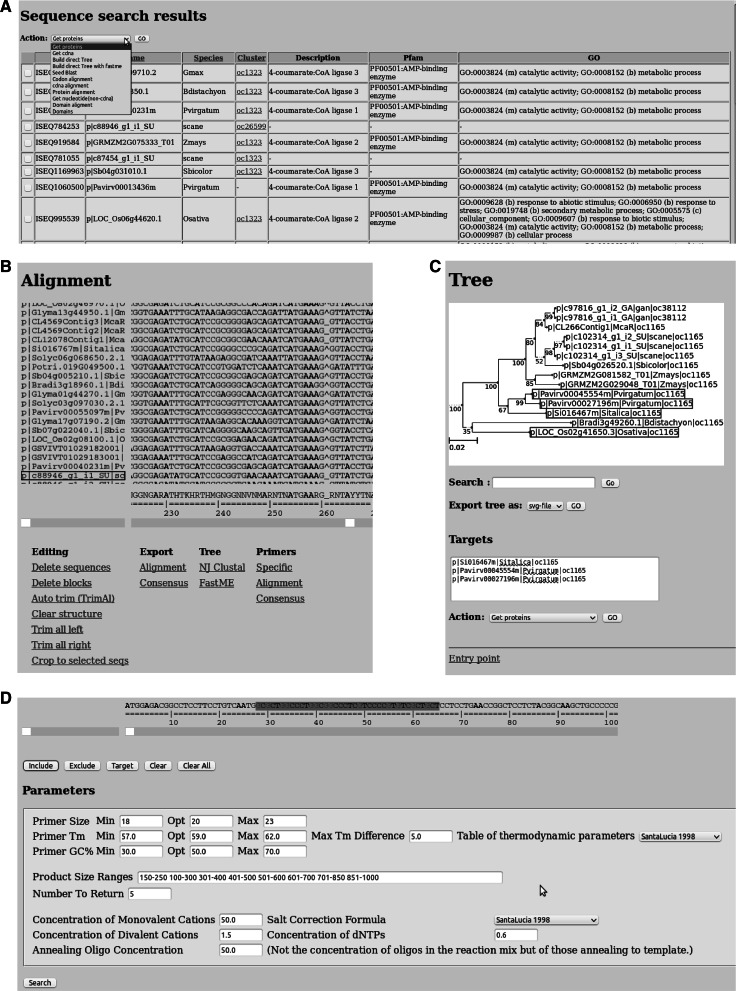
Screenshots of the orphan crop browser. **a** Sequence search results table has dropdown menu through which the user is capable of performing several actions on (a subset of) the retrieved sequences. **b** The interactive alignment module enables users to edit alignments. **c** Tree view module enables user to select sequences and perform several actions. **d** The interface to the primer3 program enables users to design primers on selected sequences

In the initial phase, users can use the text-search module to search for sequences in the database using the IDs, names, annotation key words, PFAM and GO-terms. In case the user knows beforehand that a particular sequence is a member of a homologous sequence cluster, the cluster can immediately be retrieved using its ID in the cluster-search module. In a successful search, a list of sequences is generated which can be selected for further actions (Fig. 2a).

### Similarity search

As an alternative for the text search, it is possible to perform blast searches with the blast-search module. In this module, users can set the e-value threshold and the maximum number of hits to return. Furthermore, it is possible to specify a limiting blast search to a specific set of species in the database. The browser supports the following blast searches: nucleotide search (BlastN), protein search (BlastP) and nucleotide-to-protein search (BlastX).

### Primer match

The primer-search module can be used both for the initial search and for testing whether primers are specific for the intended target (see below). In the primer-match module, primer pairs can be provided that will be searched for a user-defined set of sequence datasets. The primer-match module first uses blast to identify candidate primer matches and then performs a Needleman Wunsch alignment (Needleman and Wunsch 1970) to refine the primer/target alignment. Users can influence the number of initial blast hits to consider and the number of mismatches in the refined alignment. Furthermore, it is possible to determine the maximum and minimum lengths of the products. The primer-match module will also search for possible products that can be amplified by the combination of forward and reverse primers or by each of them independently.

### Gathering related sequences

In order to perform phylogenetic analysis, users need to gather a set of related sequences if no initial blast search was performed. One way to identify these sequences is by using the pre-defined homologous sequence cluster information (when available). Alternatively, the user can perform a sequence similarity search (blast) against the database with a selected set of sequences using the seed-blast module. In the seed-blast module, users can restrict the search to a specific set of species and determine the maximum number of hits in total and per species. In addition, users can adjust the minimum identity, alignment coverage and e-value thresholds for hits. The availability of the seed-blast module provides a solution for gathering related sequences to users that do not have the resources for large-scale clustering of highly similar sequences.

### Alignments

In the browser, users can construct protein, nucleotide and codon alignments. Codon alignments are generated by first creating protein alignments and then replacing the amino acids by the corresponding codons on the coding sequences. Alignments are displayed in a custom-built interactive alignment viewer (Fig. 2b) written in JavaScript. For nucleotide alignments, the viewer will display the consensus sequences at the bottom.

If available, the gene structure underlying the coding regions is superimposed onto the alignment. In protein alignments, the intron positions are indicated by placing the phase of the intron (Long et al. 1995) before the amino acid of which the corresponding codon is preceded or interrupted by the intron. The phase of intron indicates whether it resides between two consecutive codons, between the first and second or between the second or third nucleotide of a codon. In nucleotide alignments, intron positions are indicated by “^” characters. Previous studies have shown intron positions within the coding regions of genes are highly conserved, even between distantly related species as Arabidopsis and rice. Therefore, users can make use of the superimposed gene structure information for detecting potential alternative splicing events in an orphan crop.

The accuracy of phylogenetic tree reconstruction can be improved by removing regions of low conservation from the multiple sequence alignment (Talavera and Castresana 2007). Therefore, the alignment viewer has the possibility to remove low conserved regions from the alignment. Low conserved regions can be removed either manually or automatically by invoking the TrimAl program (Capella-Gutierrez et al. 2009).

### Phylogenetic tree module

Phylogenetic trees can be generated either by invoking ClustalW2 (Larkin et al. 2007) with a single click or by first configuring and then launching FastME (Desper and Gascuel 2002). The generated trees are rendered on the background by ETE2 (Huerta-Cepas et al. 2010) and displayed in a custom viewer that allows users to select individual or groups of sequences by clicking on the respective terminal or internal nodes (Fig. 2c). In this way, users can easily select potential targets through manual inspection of the phylogenetic tree and perform further analysis. The phylogenetic tree can be exported in PNG and SVG image formats. Additionally, users can export the tree in a Newick format, which can be opened by tree-rendering programs.

### Primer design

In order to aid researchers in confirming or quantifying transcripts, the browser provides the possibility of designing primers. There are modules for three different primer-design modes in the browsers: (1) the specific primer mode can be used to design primers for individual sequences (Fig. 2d). Intuitively, the specific mode should be used once a target gene has been identified. The specific primer-design module is simply a custom-build interface to the primer3 (Untergasser et al. 2012) program. Users can use the mouse for selecting which regions must be included in or excluded from the primer search. (2) The consensus mode is an option of the same interface as the specific mode but should be used to design primes based on the consensus sequence of an alignment. The purpose of this mode is to construct primers for perfectly conserved regions on the alignment. (3) The alignment mode can be used for designing degenerate primers. The degenerate primers can be used in the case no target sequences were identified after inspecting the phylogenetic tree. The gene might still be present in the species of interest but overlooked due to insufficient sequencing or incorrect assembly. By using the alignment of several species, users can design degenerate primers for an in-depth search for the presence the target gene in the orphan crop of interest.

Predicted primer pairs are presented in a sortable table, and the amplified gene fragments can be visualized on the sequences/alignment. This allows users to make a selection and to export it for further use. The primer-match module also enables users to check the specificity of the selected primer pairs for the desired target sequence, by checking whether they may recognize other regions and consequently result in (less specific) amplification of fragments different from the target sequence. This is an important feature to guarantee the best selection of primer pairs for amplification of your target.

### Orphan Crops Browser in action

To evaluate the applicability and accuracy of our developed browser in the search of candidate orthologues in orphan crops, a case study is presented. Given the importance of miscanthus for second-generation biofuels research and the limited amount of sequence information currently available, we chose this crop as the orphan crop of interest. The advance of second-generation biofuels mainly depends on overcoming the natural recalcitrance of the cell wall matrix to deconstruction, to directly improve the processability of lignocellulose feedstocks. Taking this feature into account, we decided to focus on finding genes involved in this process. Identifying the key genes involved in lignin biosynthesis, and correlating their expression to the content and composition on the secondary cell walls is an essential step to improve saccharification efficiency. For example, molecular engineering of key genes in lignin biosynthesis has already shown to improve the release of glucose from cell walls of switchgrass and sugarcane, without affecting plant growth, fertility or biomass yield (Jung et al. 2012; Saathoff et al. 2011). Lignin is a structural component of secondary cell walls, providing firmness and safeguard an operational vascular system, while hemicelluloses are polysaccharides able to covalently link with cellulose, lignin and some pectins, granting strength to the cell walls (Samuel et al. 2011). The accumulation of all cell wall constituents throughout plant growth and development hinders the accessibility of cellulose to hydrolyzing enzymes, thus compromising the release of sugars for cellulosic ethanol production. Finally, we choose to use sequence information from the plant species most closely related to miscanthus for the search of orthologous genes.

### Selection of *M. sinensis* genotypes

**Fig. 3:**
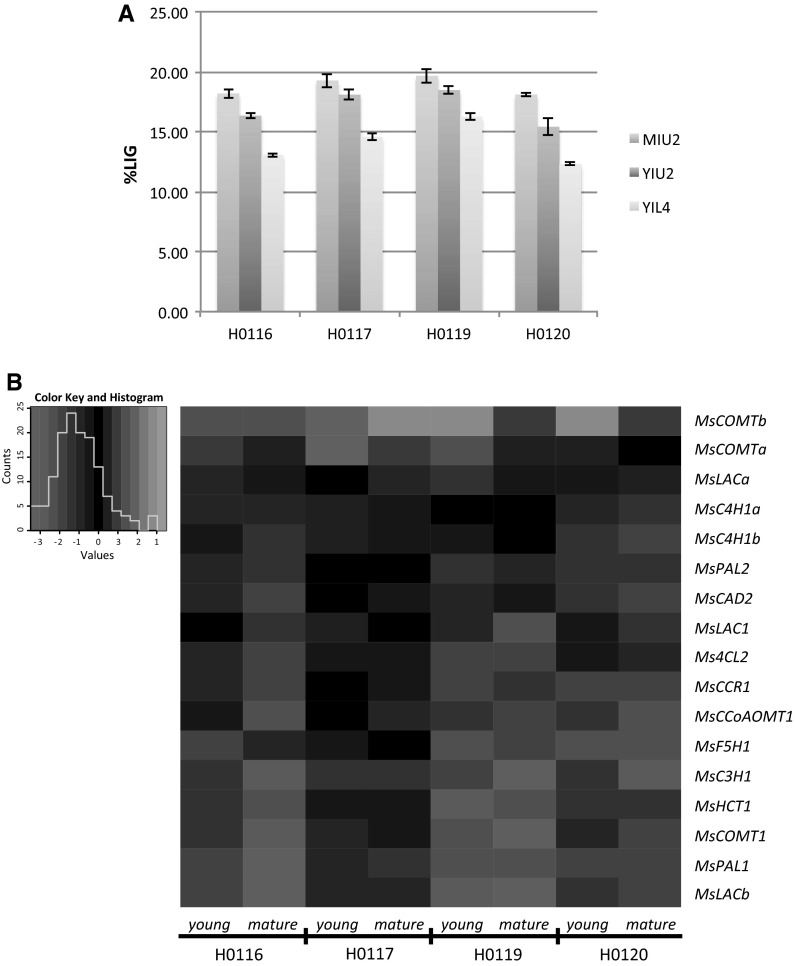
Lignin content and gene expression of studied internode sections of miscanthus. **a** Lignin cell wall content (%LIG) of selected internode segments from four *Miscanthus sinensis* genotypes (H0116, H0117, H0119 and H0120). MIU, up section of mature internode; YIU, up section of young internode; YIL, low section of young internode. **b** Heat map of cross-comparison of miscanthus lignin genes expressed in tested internode sections based on hierarchical clustering. *Each pair of columns* represents one of the studied genotypes, and each row represents a lignin gene. Down-regulated genes are indicated in *red*, while up-regulated genes in *green*. The young and mature denominations in the map correspond to the gene expression measured on YIU2 and MIU2 sections relative to the expression on YIL4, respectively. (Color figure online)

In order to identify key genes in the lignin biosynthesis in *Miscanthus sinensis*, genotypes contrasting to secondary cell wall composition were selected from a collection of approximately 120 *M. sinensis* accessions, located at Wageningen University and Research Centre (WUR). Ten stems were harvested at mature stage, from two individual plants per plot and pulled together for quantification of lignin, hemicellulose and cellulose contents (data not shown). Out of this set, four genotypes differing in cell wall properties (Table 1) were selected for a gene expression study using stem materials collected at two stages of development. Shoots were taken during the vegetative stage (<1 m height) and the generative stage (early flowering). Three internodes (i.e., internodes 2, 3 and 4 above the soil) were collected per shoot and divided into three sections, which were used for further analyses. They are referred to as up, middle or low section. The two extreme sections of each internode (up and low sections) were used to measure lignin content. Three stem samples widely differing in maturation were finally chosen for gene expression analyses for each of the four genotypes (Supplemental Table S1; Fig. 3a). The lowest lignin content was found as expected in young plants, at the meristematic region of the youngest internode (YIL4), while the highest content was observed in mature stems, in the top section of the first formed internodes, a region enriched of cells containing a mature cell wall (MIU2). In order to evaluate gene expression during stem lignification, we also included the most lignified segment from young plants (YIU2).

**Table 1:** Stem biochemical composition of four selected *Miscanthus sinensis* genotypes

Genotype	%LIG	%HEM	%CEL
H0116	13.8	28.5	40.5
H0117	13.8	30.1	42.2
H0119	12.8	26.7	46.6
H0120	15.2	29.0	39.8

%LIG, lignin; %HEM, hemicellulose; %CEL, cellulose

### Selection of candidate plant species and lignin genes

Based on our orthology predictions, the best candidate species to base the prediction of miscanthus orthologous genes were selected. Using *Vitis vinifera* as outgroup species, a total of 180 OrthoMCL clusters were identified, in which all monocot species and *V. vinifera* were represented only one time. The concatenated codon alignment of the sequences in these clusters consisted out of 146,199 sites. Using the jModelTest (Posada and Crandall 1998), we determined (based on the BIC criterion) that the most appropriate substitution model to calculate a maximum likelihood tree with the alignment was GTR+G with four rate categories. The generated maximum likelihood tree, displaying 100 % bootstrap support at all nodes, confirmed sugarcane (*Saccharum officinarum*) as the closest evolutionary-related species to miscanthus (Fig. 1b). Sugarcane and miscanthus belong to the *Saccharinae* group, and their close phylogenetically relationship has even instigated introgression of miscanthus germplasm into sugarcane to confer better growth under sub-optimal temperature (Lam et al. 2009). The high similarity between the genomes of both species has also been shown through the assembly of the first genetic map of miscanthus, when genetic markers from sugarcane were efficiently used to target specific regions in the *M. sinensis* genome (Swaminathan et al. 2012). Considering the recent works from Mazzafera’s group (Bottcher et al. 2013; Cesarino et al. 2013), we searched for miscanthus orthologues using key genes known to operate during the lignification of sugarcane stems (*SofLAC*, *Sh4CL2*, *ShC3H1*, *ShC4H1*, *ShCAD2*, *ShCCoAOMT1*, *ShCOMT1*, *ShF5H1* and *ShPAL1*). Sorghum shares a high level of collinearity with miscanthus (Kim et al. 2012; Ma et al. 2012; Swaminathan et al. 2012) and sugarcane (Wang et al. 2010) genomes, which makes the sorghum genome an ideal template for comparative genomic studies with these species. However, since currently most knowledge on lignification is described for maize, we chose to perform a translational research analysis from this model crop to miscanthus (Lawrence and Walbot 2007). Analysis of a maize dataset (Sekhon et al. 2013) resulted in the identification of differentially expressed genes possibly involved in lignification in the two analyzed internode fractions (V5_first internode and V9_fourth internode). Considering that the higher expression of these genes was mainly observed in the younger internode, we screened for genes involved in the lignin biosynthesis pathway showing higher expression in this sample (V5_first internode). In this way, eight candidates from maize (*ZmCCR1*, GRMZM2G141026, GRMZM2G140996, GRMZM2G035584, GRMZM5G842071, GRMZM2G447271, and both GRMZM2G081582 and GRMZM2G029048) were selected, two of them that showed homology to sugarcane genes (*ShHCT1* and *ShPAL2*). These two sugarcane genes were not initially added in our analysis, since they were considered not to have a role on stem lignification in sugarcane; however, we still decided to include them in our study since they are up-regulated in young maize internodes. A list with all used candidate genes in our analysis is summarized in Table 2.

**Table 2:** Lignin candidate genes from sugarcane, maize and their putative *Miscanthus sinensis* orthologues used in this work

Family; description	Maize gene ID	Sugarcane gene ID	Sugarcane orthologues in OCB^a^	*Miscanthus* gene ID	*Miscanthus* orthologues in OCB	References
4CL; 4-coumarate:CoA ligase 2	GRMZM2G055320	*Sh4CL2*	c88946_g1_i1_SU c88946_g1_i2_SU	*Ms4CL2*	CL12078Contig1	Bottcher et al. (2013) Shangguan et al. (2013) Zhang et al. (2014)
C3H; coumarate-3-hydroxylase	GRMZM2G140817	*ShC3H1*	c70890_g1_i1_SU	*MsC3H1*	CL5399Contig1 CL5399Contig2	Bottcher et al. (2013)
C4H; cinnamate-4-hydroxylase	GRMZM2G139874 1st cluster	*ShC4H1*	c94596_g1_i1_SU	*MsC4H1a*	CL585Contig3 m454_isotig05388 m454_isotig05386 MU_comp33105_c1_seq10	Bottcher et al. (2013) Courtial et al. (2013) Shangguan et al. (2013)
	2nd cluster	NA	NA	*MsC4H1b*	CL585Contig2 MF_comp34800_c0_seq1 MF_comp34800_co_seq3	
CAD; cinnamyl alcohol dehydrogenase	GRMZM5G844562	*ShCAD2*	c91614_g1_i2_SU c91614_g1_i1_SU	*MsCAD2*	CL3287Contig1 CL3287Contig2 CL3287Contig3 CL3287Contig4	Courtial et al. (2013) Tanaka et al. (2014) Zhang et al. (2014)
CCoAOMT; S-adenosyl-l-methionine-dependent methyltransferases superfamily protein	GRMZM2G099363 GRMZM2G127948	*ShCCoAOMT1*	c107456_g2_i1_SU	*MsCCoAOMT1*	CL4519Contig1 CL24843Contig1	Bosch et al. (2011) Bottcher et al. (2013) Courtial et al. (2013) Li et al. (2013) Wen et al. (2014) Zhang et al. (2014)
CCR; cinnamoyl coa reductase	GRMZM2G131205 (*ZmCCR1*)	NA	NA	*MsCCR1*	CL5621Contig1 CL5621Contig2 CL5621Contig3 CL23415Contig1	Khan et al. (2010) Bosch et al. (2011) Tamasloukht et al. (2011) Courtial et al. (2013) Liseron-Monfils et al. (2013) Tanaka et al. (2014) Zhang et al. (2014)
COMT; O-methyltransferase family protein	AC196475.3_FGT004	*ShCOMT1*	c96304_g1_i2_SU c96304_g1_i1_SU	*MsCOMT1*	CL3733Contig1 CL3733Contig2 m454_isotig10910 m454_isotig10911	Bottcher et al. (2013)
	GRMZM2G141026	NA	NA	*MsCOMTa*	MU_comp25105_c0_seq2 MF_comp27777_c0_seq2 MF_comp27777_c0_seq4	Bosch et al. (2011) Courtial et al. (2013) Meihls et al. (2013)
	GRMZM2G140996	NA	NA	*MsCOMTb*	MU_comp504097_c0_seq1	Bosch et al. (2011) Courtial et al. (2013) Meihls et al. (2013)
F5H; ferulic acid 5-hydroxylase	AC210173.4_FGT005	*ShF5H1*	c101416_g2_i1_SU	*MsF5H1*	CL8875Contig1	Bottcher et al. (2013)
HCT; hydroxycinnamoyl-CoA shikimate/quinate hydroxycinnamoyl transferase	GRMZM2G035584	*ShHCT1*	c93886_g2_i1_SU	*MsHCT1*	CL17112Contig1	Courtial et al. (2013)
LAC; Laccase	GRMZM2G305526	*SofLAC*	NA	*MsLAC1*	CL12102Contig1	Cesarino et al. (2013)
	GRMZM5G842071	NA	NA	*MsLACa*	CL15280Contig1	Courtial et al. (2013)
	GRMZM2G447271	NA	NA	*MsLACb*	CL3689Contig1	Courtial et al. (2013)
PAL; PHE ammonia lyase	GRMZM2G074604	*ShPAL1*	c100670_g1_i1_SU	*MsPAL1*	CL266Contig4 MF_comp35858_c1_seq3 m454_isotig15917	Bosch et al. (2011) Bottcher et al. (2013) Courtial et al. (2013) Shangguan et al. (2013) Tanaka et al. (2014) Zhang et al. (2014)
	GRMZM2G081582 GRMZM2G029048	*ShPAL2*	c102314_g1_i3_SU	*MsPAL2*	CL266Contig3	Bosch et al. (2011) Bottcher et al. (2013) Courtial et al. (2013) Liseron-Monfils et al. (2013) Shangguan et al. (2013)

The miscanthus gene ID was given according to the nomenclature adopted in sugarcane whenever possible

^a^Sugarcane orthologous are only provided if they were used as the starting point for tree construction

### Identification and evaluation of *Miscanthus sinensis* orthologous genes

After selecting the lignin candidate genes from sugarcane and maize, a two-step approach was adopted for the identification of miscanthus orthologues (Fig. 1c). First, a S*equence Search* was performed for each candidate gene ID separately, resulting in the identification of its correspondent orthologous group. All sequences from each group were used to construct a phylogenetic tree, which allowed the identification of single or multiple miscanthus orthologous candidate genes. Overall, multiple miscanthus orthologous sequences were generally found for each candidate gene, with a few exceptions. Orthologous sequences from *M. x giganteus* were found for seven candidate genes and were kept in the analysis to support the alignments (data not shown). All generated phylogenetic trees are shown in Supplemental Figures S1A-P. As second step, the selected single or consensus miscanthus sequences were used as templates for the design of specific primers and further checked in silico for efficiency. Preceding the use of primers for quantitative gene expression analyses, the specificity of each pair of primers was also confirmed by sequencing of the amplified gene fragment (for primers list, see Supplemental Table S2).

To evaluate how lignin accumulation would be correlated with gene expression, the first step was the analysis of how gene expression variation associates with distinguishable developmental stages in early plant growth. A cross-comparison was performed within the young plant, focusing on the most mature section of the oldest internode (YIU2) and the meristematic section of the youngest internode (YIU2 × YIL4). Lignification was more prominent in YIU2 (Fig. 3a), whereas the lignin genes showed higher expression levels in YIL4 for all four genotypes (Fig. 3b). Although the down-regulation of lignin genes was less obvious in the genotype H0117, for the other three genotypes only two to three genes showed up-regulation. In all genotypes, the *MsCOMTa* and *MsCOMTb* genes were the only genes with increased expression following tissue lignification. Since lignification is a process linked to tissue maturity, we decided to confirm these results in a more contrasting scenario, by carrying out a second comparison on plants in different developmental stages (MIU2 × YIL4). As expected, the down-regulation of lignin genes was even more pronounced in this stage enriched of cells containing mature cell walls. In addition, the expression of the *MsLACa* gene slightly increased in genotype H0120 (Supplemental Table S3).

## Discussion

Here we presented the Orphan Crops Browser, a novel user-friendly tool for the identification of orthologous genes in orphan crops and to assist molecular breeding of these species. We have illustrated its capabilities by identifying and studying key genes involved in secondary cell wall lignification in *M. sinensis*, an important bioenergy feedstock candidate, using knowledge from closely related C4 plants. Some online tools exist that can perform the tasks of some of the individual modules in the browser. However, the browser enables the users to perform all those tasks without constantly needing to transfer data between Web pages. In contrast to Trinotate-web, the Orphan Crops Browser provides a graphical user interface that simplifies the database construction, which is needed for exploring the data. As the Orphan Crops Browser was built for the purpose of target gene identification, it is sufficient to import a de novo transcript together with a set of species with annotated genomes. Therefore, the Orphan Crops Browser does not perform large-scale annotations of de novo transcriptomes in the same manner as Trapid.

In contrast to the Orphan Crops Browser, neither Trapid nor Trinotate-web has the possibility to design and test specific and degenerate primers. These tools do not provide a solution for the situation in which a target sequence cannot be found due to, for instance, insufficient sequencing depth. Our tool overcomes this problem, by providing users with the possibility of designing degenerated primers for confirming the presence or absence of a particular gene in the orphan crop. This is particularly useful in those cases where closely related species contain certain genes that were not assembled in the orphan crop due to, for instance, low expression levels. The user friendliness of the Orphan Crop Browser will especially be useful for researches with limited bioinformatics knowledge.

In the miscanthus case study, we were able to identify 17 orthologous genes in *M. sinensis* having a putative role in lignification with the help of the Orphan Crops Browser. In general, our results showed a down-regulation of lignin genes in stem tissues in parallel with lignin accumulation, suggesting that gene expression decreases as cell wall matures. Recent works support our findings, such as the research of Sekhon et al. (2013), which showed through a microarray analysis that in maize tissues the highest expression for the majority of lignin genes is found in the most immature organs. This indicates the most active developmental processes on secondary cell wall formation and lignification starts when cells are young. Results of a detailed transcriptome analysis made by Zhang and collaborators (Zhang et al. 2014) using elongating maize internodes showed that the peak of lignin genes expression is found in the internode section showing cell division and active elongation, with expression being no longer detected when elongation stops and lignin continues to accumulate. In sugarcane, with the exception of *SofLAC*, the determinant criterion used to select genes to have a key role in the lignin pathway was the association of gene expression in stem tissues with lignin deposition (Bottcher et al. 2013; Cesarino et al. 2013). Analysis of two maize internodes with contrasting levels of lignification showed that expression of *MsCCR1*, *MsCOMT1*, *MsCOMTa*, *MsCCoAOMT1*, *MsPAL1* and *MsPAL2* was higher in the most lignified internode, except for the *MsCOMTb* gene (Bosch et al. 2011). Most probably, meristematic and elongating internode sections produce more lignin monomer components, more important in the first steps of lignification. At later stages, lignin genes would reduce their expression before a cell reached maturity, taking advantage of the stability of enzymes and storage monolignols to continue the stem lignification. Increased lignification during post-harvest has been already observed in asparagus spears (Hennion et al. 1992), which validates the idea of lignification occurring in the absence of an active gene expression, possibly due to high stability of lignin biosynthetic enzymes. The formation of the lignin polymer requires the synthesis of different monolignols, mainly p-coumaryl, coniferyl and sinapyl alcohols, which form the basis of the phenylpropanoids p-hydroxyphenyl (H), guaiacyl (G) and syringyl (S) units, respectively. Understanding their biosynthesis is essential to be able to improve plant biomass composition (Boerjan et al. 2003; Li and Chapple 2010). In the case of *M. sinensis*, only *MsCOMT* genes were up-regulated in mature tissues, and that may indicate that the need for syringyl (S) units is higher during the last stages of stem lignification. Cell walls rich in S-units are known to be less recalcitrant to saccharification, in comparison with G-rich cell walls (Huntley et al. 2003). This finding makes *MsCOMT* genes interesting genetic targets to improve intrinsic properties of cell walls and to develop better feedstocks for second-generation biofuels. Altogether, the browser revealed to be versatile and efficient in the identification lignin genes in miscanthus and primer design for expression studies. We have shown that the OCB browser is an effective tool to identify specific breeding targets in orphan crops and assist molecular breeding.

## Materials and methods

### Web browser

The Web browser was constructed using the Django framework for Python, in combination with HTML and JavaScript. All biological data are stored in MySQL databases. Several features of the browser use external programs (see below).

### Primer design

Specific primer pairs are predicted using the Primer3 (Untergasser et al. 2012) command line program. Degenerate primer pair predictions start by searching the nucleotide alignment of all potential primers with predefined lengths and maximum degeneracy lying with specific distance. For each degenerate primer pair, the following procedure is respected: First, all possible primer sequences are determined from the degenerate sequences. Next, the melting temperature is calculated for each primer sequence using the oligotm function (oligotm.h from the Primer3 source code). All primers with melting temperatures outside the user-defined range are removed, including the ones predicted to form homo-dimers according to the dpal function (dpal.h) from Primer3 or that have high or low GC content. From the remaining set of primers, pairs that potentially form hetero-dimers according to the dpal function are discarded. Finally, primer pairs that can amplify all sequences are reported to the user.

### Primer matching

All possible forward and reverse primer sequences are determined for primers containing degenerate nucleotides. Initial matches to the database sequence are obtained through BlastN (Altschul et al. 1997) searches with user-defined parameters. Primers with significant blast hits are realigned to the corresponding target sequences using the Needleman Wunsch alignment (Needleman and Wunsch 1970). Primers pairs for which each of the NW alignments meets user-defined requirements and is separated by a predefined distance are reported back to the user.

### Alignments

All alignments are made using the MUSCLE version 3.5 (Edgar 2004). Codon alignments are generated from the protein alignments by substituting each amino acid with its corresponding codon and extending each gap to three.

### Phylogenetic trees within the browser

Neighbor joining trees are constructed using ClusterW2 (Larkin et al. 2007) or FastME (Desper and Gascuel 2002). The support for each node in the tree is determined through bootstrap analysis. The trees are subsequently rendered using the ETE package (Huerta-Cepas et al. 2010) version 2.

### Case study using *Miscanthus sinensis*

The following short read sequences were downloaded from the NCBI SRA repository: *Saccharum officinarum* (SRR89062); *M. sinensis* varieties Gross Fontaine (SRX131848) and Udine (SRX131681); *M. sinensis* 454 sequences (SRR916887); *M. x giganteus* (SRP017791).

### De novo transcriptome assembly

All Illumina reads were trimmed using prinseq-lite 0.20.4 (http://prinseq.sourceforge.net/). The cleaned reads were assembled using Trinity with the minimum Kmer coverage parameter set to 2. Separate assemblies were performed for the Gross Fontaine and Undine *Miscanthus* varieties. The *M. sinensis* 454 reads were assembled into contigs using Newbler version 2.6 (http://www.454.com/products/analysis-software/). An overall *M. sinensis* transcriptome was generated by merging the Gross Fontaine, Udine and 454 assemblies using the TGICL package (Pertea et al. 2003).

### Annotation of assembled contigs

All contigs were searched against the non-redundant protein database from NCBI using BlastX (e-value 1e-5). All sequences for which the best hit was a non-plant protein were discarded. The remaining sequences were annotated by importing the blast results into Blast2GO (Conesa et al. 2005). Open reading frames were predicted using ESTscan that specifically trained for each species as follows: First, the assembled contigs were realigned to their best blast hit using exonerate (Slater and Birney 2005). All alignments were required to be devoid of frame shifts and to contain at least 80 % of the residues of the known protein. Those contigs for which the exonerate alignments met the criteria and that contained open reading frames (from start to stop) including UTR sections were used as training sequences for ESTscan (Iseli et al. 1999).

### Plants with sequenced genomes

The protein sequences, coding sequences, gene structure and annotation data were downloaded from Phytozome for the following species: *Arabidopsis thaliana*, *Glycine max*, *Vitis vinifera*, *Sorghum bicolor*, *Panicum virgatum*, *Zea Mays*, *Oryza sativa*, *Brachipodiumdistachyon* and *Setaria italica.*

### Function protein domains

Functional protein domains (PFAM) were predicted using pfamscan.pl (ftp://ftp.ebi.ac.uk/pub/databases/Pfam/Tools/).

### Orthologous groups

An all-versus-all protein similarity search was performed using BlastP (1e-5). Orthologous groups were predicted from the blast output using OrthoMCL version 2.9 (Li et al. 2003).

### Phylogenetic tree

OrtholoMCL clusters were filtered and only the clusters in which all monocot species in our database and *Vitis venifera* were represented by only one sequence. The protein sequences within each individual cluster were aligned using MUSCLE. The resulting protein alignments were converted to codon alignments and subsequently concatenated into a single large alignment using in-house scripts. jModelTest version 2.1.7 (Posada and Crandall 1998) was used for determining the appropriate substitution model for construction maximum likelihood trees. A maximum likelihood tree was constructed from the concatenated alignment using PhyML (Guindon et al. 2010). The resulting tree was rooted and visualized using ETE2 (Huerta-Cepas et al. 2010).

### Plant material

The four *Miscanthus sinensis* genotypes, known as H0116, H0117, H0119 and H0120, were collected for both biochemical and gene expression analyses, from two independent plants in the field. In average, 20 shoots were harvested, chopped and air-dried at 70 °C for 48 h, and subsequently ground to a fine powder using a ball mill (Retsch, Haan, Germany) for biochemical compositional analyses. In parallel, 10 shoots were harvested at the same time and transferred to liquid nitrogen and milled (Mill Pulverisette 14, FRITSCH) for RNA extraction.

### Compositional analyses

Neutral detergent fiber (NDF) components were determined as described by (Torres et al. 2013), and lignin content was quantified by the acetyl bromide method using the cell wall residue obtained from the NDF treatment as described in (De Souza et al. 2015).

### RNA manipulation

Total RNA was extracted from miscanthus internode sections using the TRIZOL reagent (Invitrogen) according to manufacturer’s instructions. RNA concentration and purity were quantified with Nanodrop measurements, and the quality of the total RNA was checked on a 1.5 % RNase-free agarose gel. After DNase treatment with the DNase I Amplification Grade kit (Invitrogen) and purification using the RNeasy Mini Kit (Qiagen), cDNA was synthesized with the iScript cDNA synthesis kit (Bio-Rad). To investigate whether the selected primers were amplifying the predicted regions defined by the Orphan Crops Browser correctly, all amplified fragments were cloned and subsequently sequenced. A pool from all cDNA samples was used as a template, and fragments were amplified using Pfu DNA Polymerase (Promega) and fused to the pENTR™/D-TOPO^®^ Gateway vector (Invitrogen). Quantitative RT-PCR was performed using a Bio-RAD detection system and the SYBR Green kit (Roche), with 3 µM specific primers, 10 ng of cDNA in a total volume of 10 μL per reaction. Two independent runs were performed as technical replicates, and samples were analyzed in triplicate per run, with the use of the actin reference gene for normalization (Straub et al. 2013b). Gene expression fold change (2^*n*^) was calculated using the −ΔΔCT method (Livak and Schmittgen 2001).

## Electronic supplementary material

Supplementary material 1 (PDF 2473 kb)

Supplementary material 2 (PDF 100 kb)

Supplementary material 3 (PDF 16 kb)

Supplementary material 4 (PDF 131 kb)

Supplementary material 5 (PDF 99 kb)
