# Supplementary material for: Orphan Crops Browser: a bridge between model and orphan crops
Source: Mol Breed. 2016 Jan 12;36:9. doi: 10.1007/s11032-015-0430-2 (PMC4710642; doi:10.1007/s11032-015-0430-2)
Supplement: Supplementary file 1 — Supplementary material 1 (PDF 2473 kb) [file 11032_2015_430_MOESM1_ESM.pdf]

Figure S1. Phylogenetic analyses generated by the Orphan Crops Browser, using codon alignments from lignin candidate genes. **A**, *Ms4CL2*; **B**, *MsC3H1*; **C**, *MsC4Ha* and *b*; **D**, *MsCAD2*; **E**, *MsCCoAOMT1*; **F**, *MsCCR1*; **G**, *MsCOMT1*; **H**, *MsCOMTa*; **I**, *MsCOMTb*; **J**, *MsF5H1*; **K**, *MsHCT1*; **L**, *MsLAC1*; **M**, *MsLACa*; **N**, *MsLACb*; **O**, *MsPAL1* and **P**, *MsPAL2*. Each entry in the trees provides the following information: gene name, according to Phytozome annotation; species name and orthologous cluster (oc) identification number. For simplicity, species names were shorten as follows: Athaliana - *Arabidopsis thaliana*; Bdistachyon - *Brachypodium distachyon*; Gmax - *Glycine max*; gan - *Miscanthus x giganteus*; Mcar - *Miscanthus sinensis*; Osativa - *Oryza sativa*; Pvirgatum - *Panicum virgatum*; Ptrichocarpa - *Populus trichocarpa*; scane - *Saccharum officinarum*; Sitalica - *Setaria italica*; Slycopersicum - *Solanum lycopersicum*; Sbicolor - *Sorghum bicolor*; Vvinifera - *Vitis vinifera*; Zmays - *Zea mays*. Whenever present, the maize and sugarcane orthologs to miscanthus were shown in the tree in bold, while the miscanthus candidates used for primer design were colored in red. Sugarcane orthologs were only highlighted in those cases where it was directly used for constructing the phylogenetic tree. For large trees, the relevant subtrees are shown. The procedure for each tree construction is explained in detail in Supplemental Table S4.

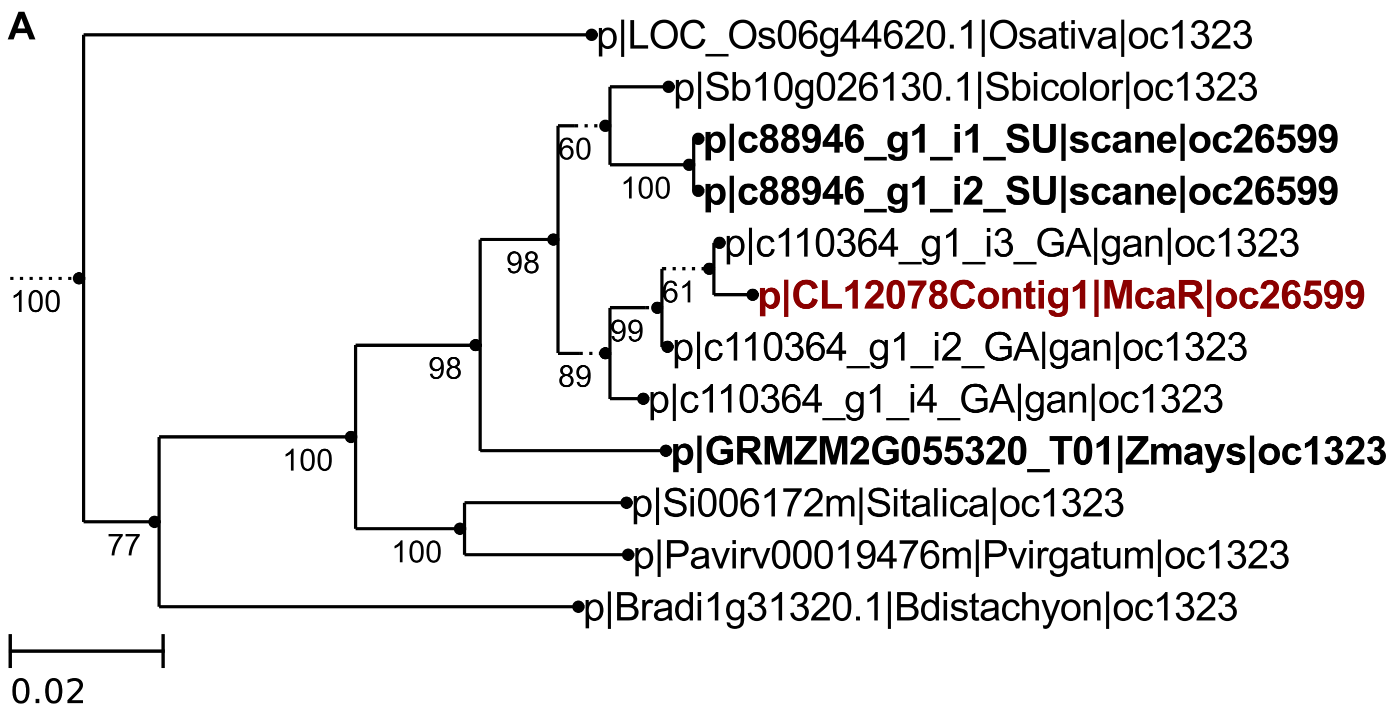

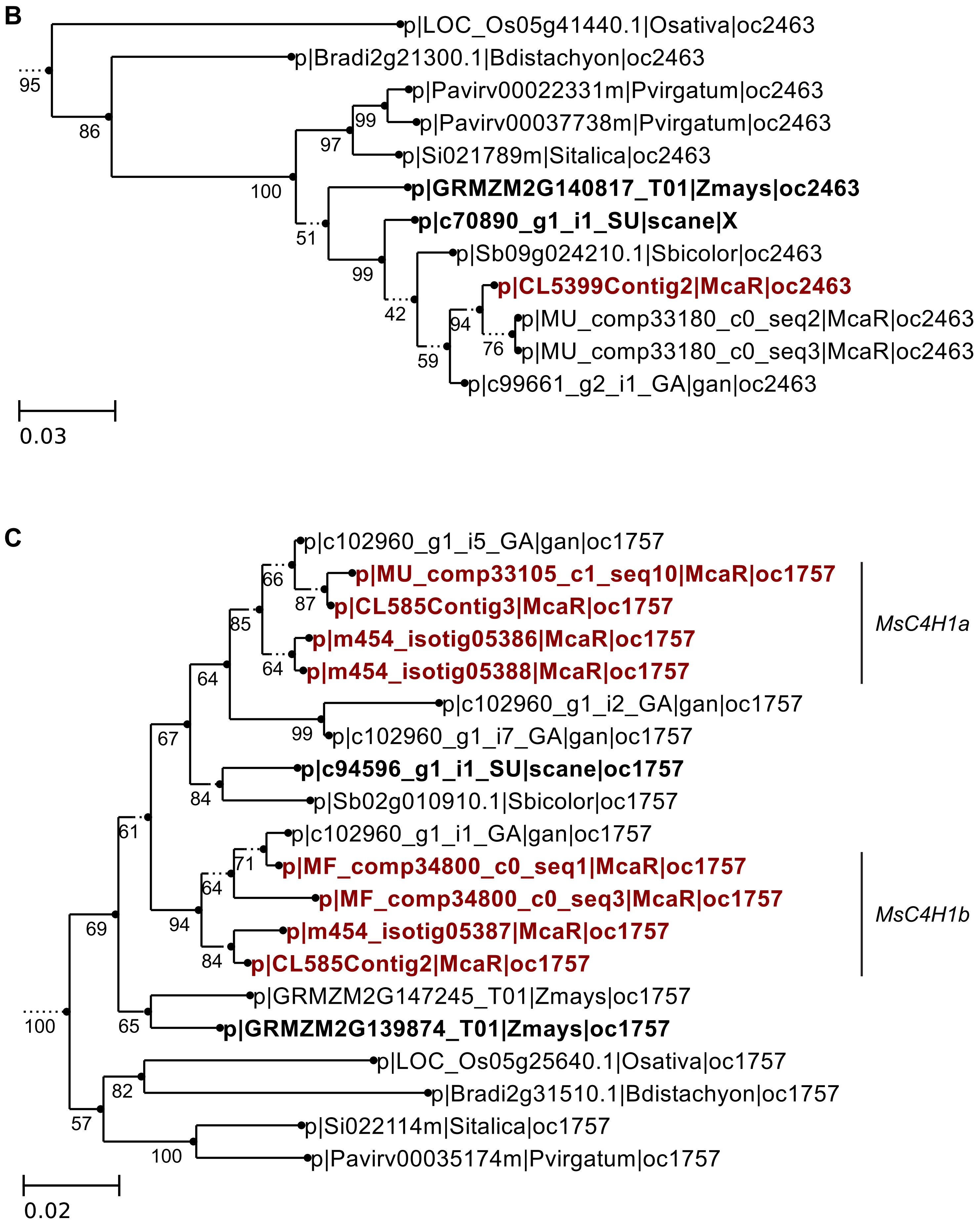
B
p|LOC_Os05g41440.1|Osativa|oc2463
p|Bradi2g21300.1|Bdistachyon|oc2463
p|Pavirv00022331m|Pvirgatum|oc2463
p|Pavirv00037738m|Pvirgatum|oc2463
p|Si021789m|Sitalica|oc2463
p|GRMZM2G140817_T01|Zmays|oc2463
p|c70890_g1_i1_SU|scane|X
p|Sb09g024210.1|Sbicolor|oc2463
p|CL5399Contig2|McaR|oc2463
p|MU_comp33180_c0_seq2|McaR|oc2463
p|MU_comp33180_c0_seq3|McaR|oc2463
p|c99661_g2_i1_GA|gan|oc2463
95
86
99
97
100
51
99
42
94
76
59
0.03
C
p|c102960_g1_i5_GA|gan|oc1757
p|MU_comp33105_c1_seq10|McaR|oc1757
p|CL585Contig3|McaR|oc1757
p|m454_isotig05386|McaR|oc1757
p|m454_isotig05388|McaR|oc1757
p|c102960_g1_i2_GA|gan|oc1757
p|c102960_g1_i7_GA|gan|oc1757
p|c94596_g1_i1_SU|scane|oc1757
p|Sb02g010910.1|Sbicolor|oc1757
p|c102960_g1_i1_GA|gan|oc1757
p|MF_comp34800_c0_seq1|McaR|oc1757
p|MF_comp34800_c0_seq3|McaR|oc1757
p|m454_isotig05387|McaR|oc1757
p|CL585Contig2|McaR|oc1757
p|GRMZM2G147245_T01|Zmays|oc1757
p|GRMZM2G139874_T01|Zmays|oc1757
p|LOC_Os05g25640.1|Osativa|oc1757
p|Bradi2g31510.1|Bdistachyon|oc1757
p|Si022114m|Sitalica|oc1757
p|Pavirv00035174m|Pvirgatum|oc1757
MsC4H1a
MsC4H1b
66
87
85
64
64
99
67
84
61
71
64
94
84
69
65
100
82
57
100
0.02

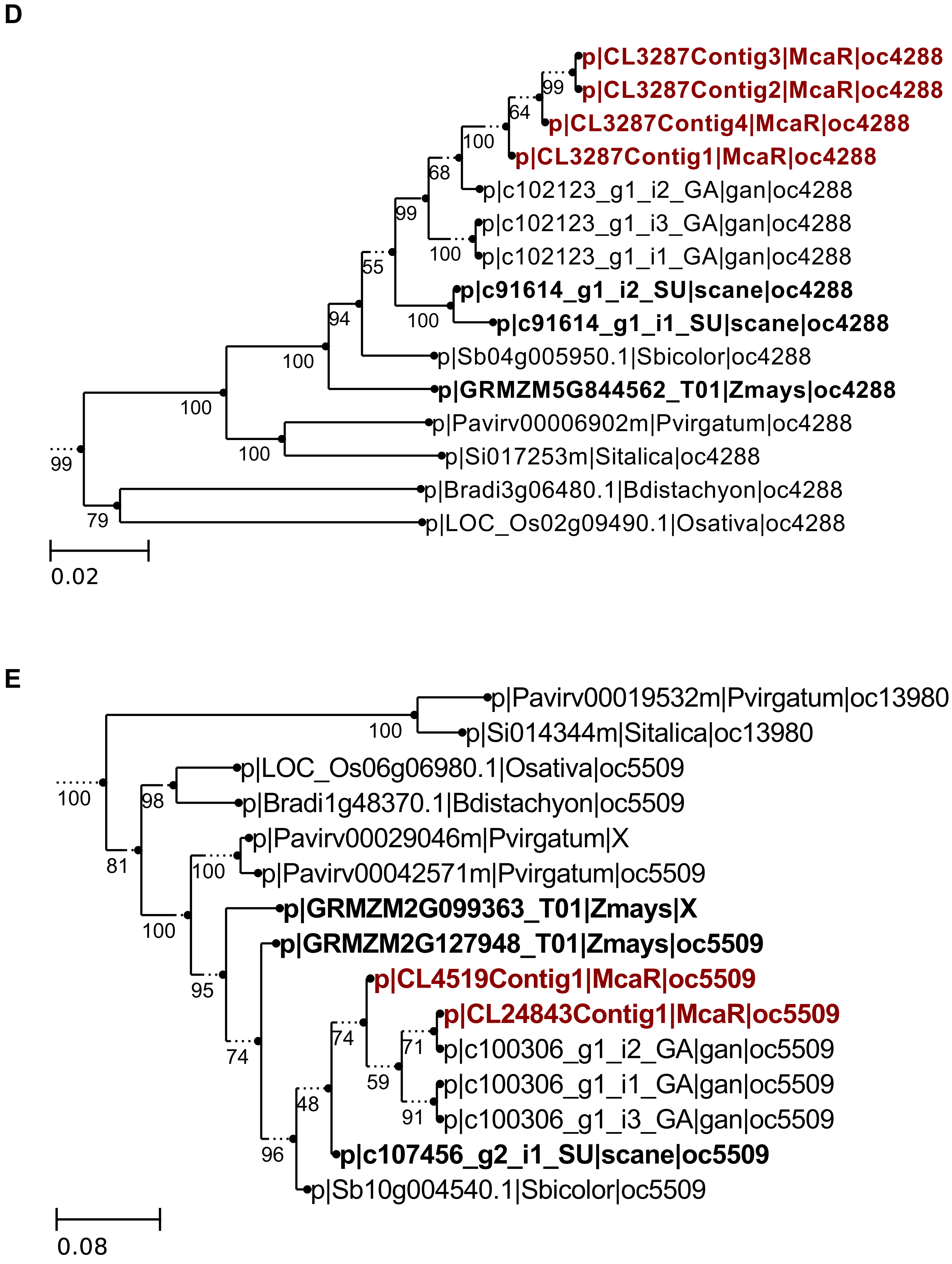
D
p|CL3287Contig3|McaR|oc4288
p|CL3287Contig2|McaR|oc4288
p|CL3287Contig4|McaR|oc4288
p|CL3287Contig1|McaR|oc4288
p|c102123_g1_i2_GA|gan|oc4288
p|c102123_g1_i3_GA|gan|oc4288
p|c102123_g1_i1_GA|gan|oc4288
p|c91614_g1_i2_SU|scane|oc4288
p|c91614_g1_i1_SU|scane|oc4288
p|Sb04g005950.1|Sbicolor|oc4288
p|GRMZM5G844562_T01|Zmays|oc4288
p|Pavirv00006902m|Pvirgatum|oc4288
p|Si017253m|Sitalica|oc4288
p|Bradi3g06480.1|Bdistachyon|oc4288
p|LOC_Os02g09490.1|Osativa|oc4288
99
64
100
68
99
100
55
100
94
100
100
100
99
79
0.02
E
p|Pavirv00019532m|Pvirgatum|oc13980
p|Si014344m|Sitalica|oc13980
p|LOC_Os06g06980.1|Osativa|oc5509
p|Bradi1g48370.1|Bdistachyon|oc5509
p|Pavirv00029046m|Pvirgatum|X
p|Pavirv00042571m|Pvirgatum|oc5509
p|GRMZM2G099363_T01|Zmays|X
p|GRMZM2G127948_T01|Zmays|oc5509
p|CL4519Contig1|McaR|oc5509
p|CL24843Contig1|McaR|oc5509
p|c100306_g1_i2_GA|gan|oc5509
p|c100306_g1_i1_GA|gan|oc5509
p|c100306_g1_i3_GA|gan|oc5509
p|c107456_g2_i1_SU|scane|oc5509
p|Sb10g004540.1|Sbicolor|oc5509
100
100
98
81
100
100
95
74
74
71
59
91
48
96
0.08

F

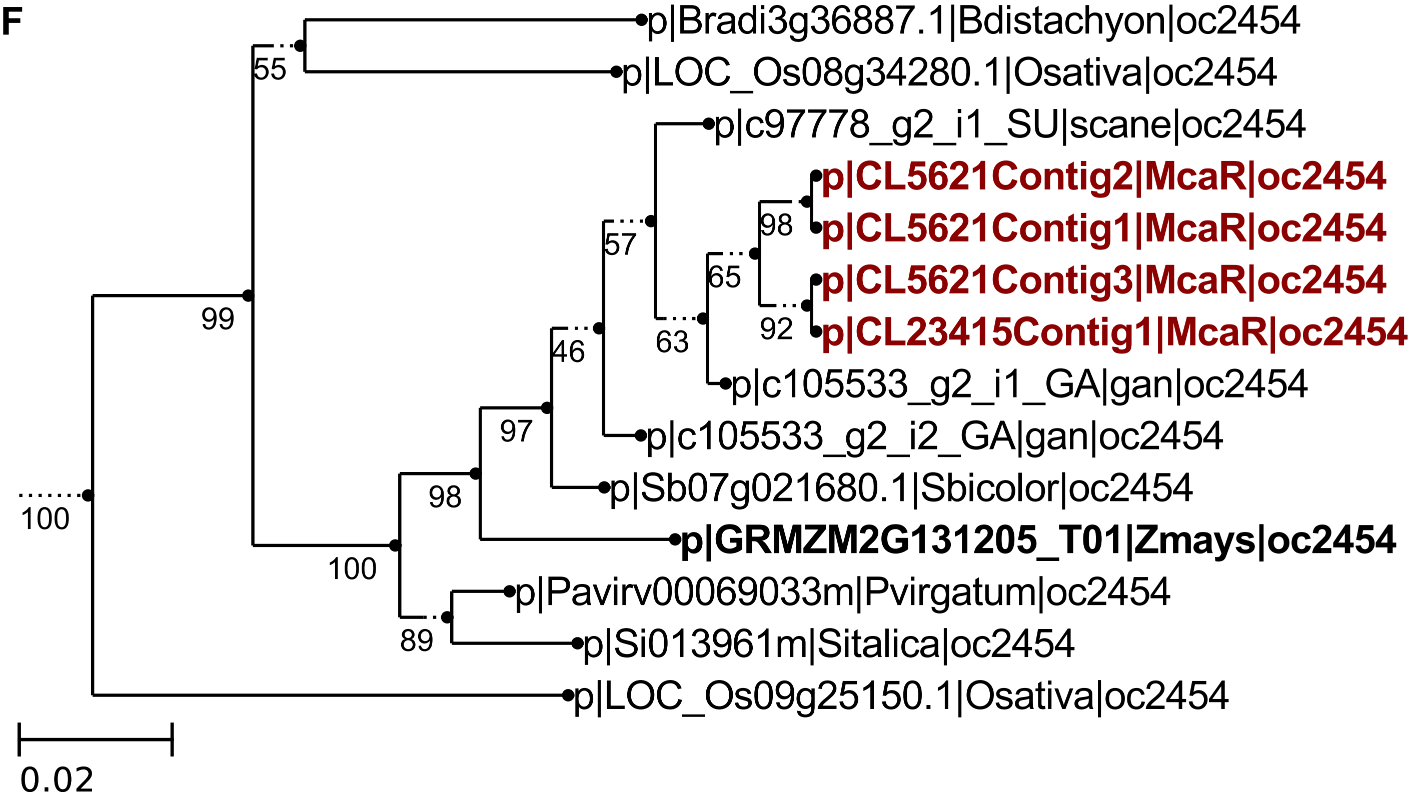
p|Bradi3g36887.1|Bdistachyon|oc2454
p|LOC_Os08g34280.1|Osativa|oc2454
p|c97778_g2_i1_SU|scane|oc2454
p|CL5621Contig2|McaR|oc2454
p|CL5621Contig1|McaR|oc2454
p|CL5621Contig3|McaR|oc2454
p|CL23415Contig1|McaR|oc2454
p|c105533_g2_i1_GA|gan|oc2454
p|c105533_g2_i2_GA|gan|oc2454
p|Sb07g021680.1|Sbicolor|oc2454
p|GRMZM2G131205_T01|Zmays|oc2454
p|Pavirv00069033m|Pvirgatum|oc2454
p|Si013961m|Sitalica|oc2454
p|LOC_Os09g25150.1|Osativa|oc2454
55
99
57
98
65
63
92
46
97
98
100
100
89
0.02

G

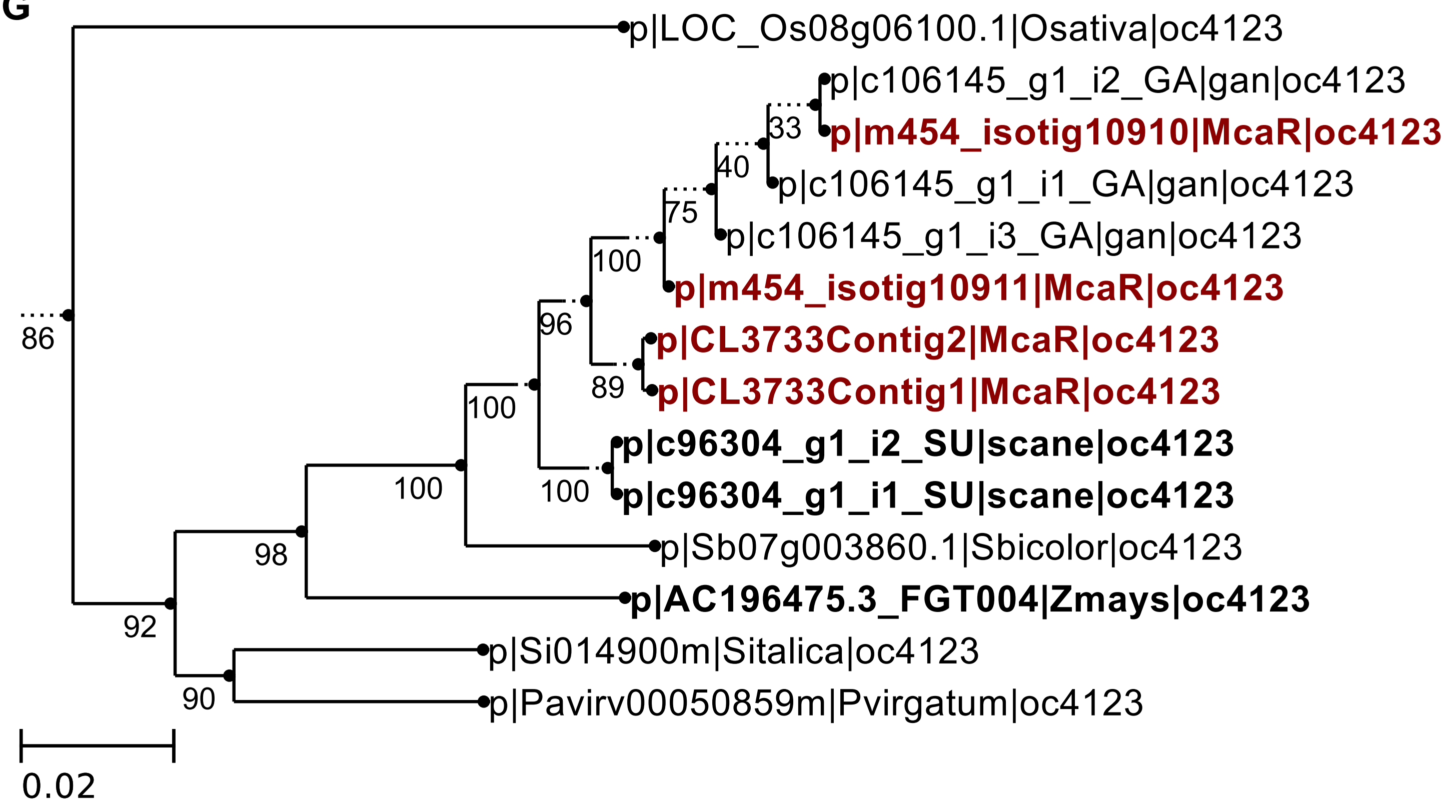
p|LOC_Os08g06100.1|Osativa|oc4123
p|c106145_g1_i2_GA|gan|oc4123
p|m454_isotig10910|McaR|oc4123
p|c106145_g1_i1_GA|gan|oc4123
p|c106145_g1_i3_GA|gan|oc4123
p|m454_isotig10911|McaR|oc4123
p|CL3733Contig2|McaR|oc4123
p|CL3733Contig1|McaR|oc4123
p|c96304_g1_i2_SU|scane|oc4123
p|c96304_g1_i1_SU|scane|oc4123
p|Sb07g003860.1|Sbicolor|oc4123
p|AC196475.3_FGT004|Zmays|oc4123
p|Si014900m|Sitalica|oc4123
p|Pavirv00050859m|Pvirgatum|oc4123
33
40
75
100
96
89
100
100
100
86
98
92
90
0.02

H

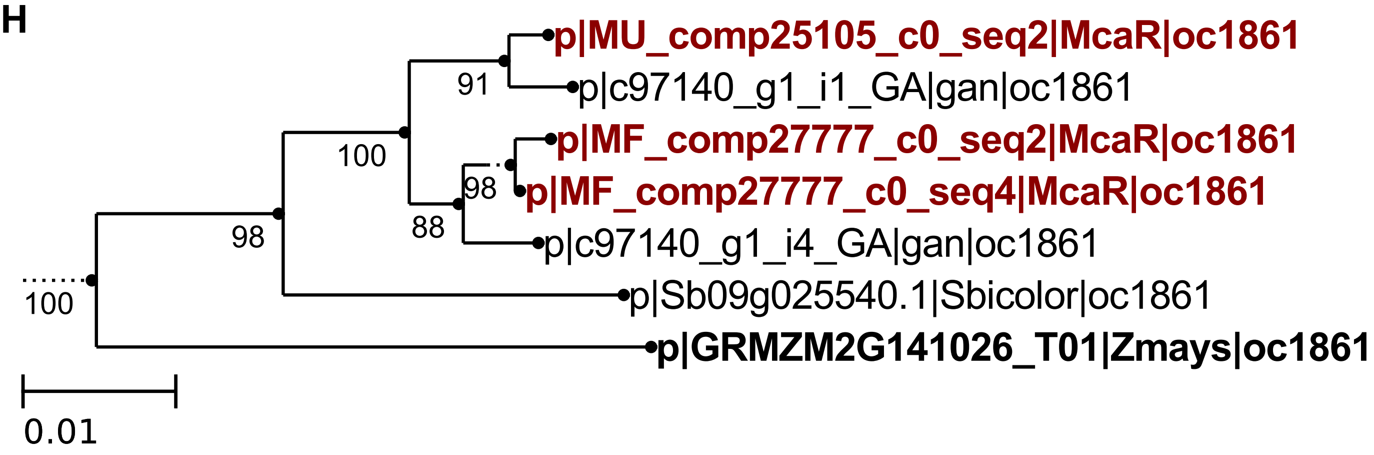
p|MU_comp25105_c0_seq2|McaR|oc1861
p|c97140_g1_i1_GA|gan|oc1861
p|MF_comp27777_c0_seq2|McaR|oc1861
p|MF_comp27777_c0_seq4|McaR|oc1861
p|c97140_g1_i4_GA|gan|oc1861
p|Sb09g025540.1|Sbicolor|oc1861
p|GRMZM2G141026_T01|Zmays|oc1861
91
100
98
88
98
100
0.01

I

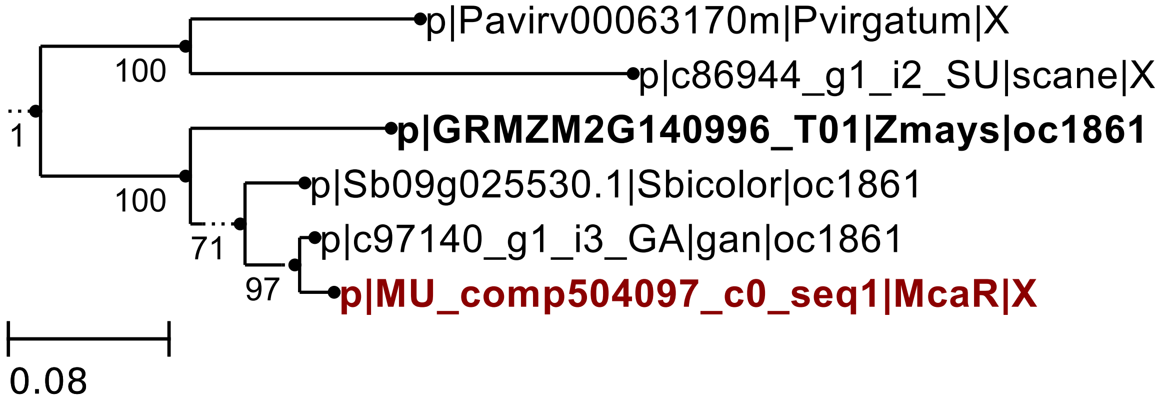
p|Pavirv00063170m|Pvirgatum|X
p|c86944_g1_i2_SU|scane|X
p|GRMZM2G140996_T01|Zmays|oc1861
p|Sb09g025530.1|Sbicolor|oc1861
p|c97140_g1_i3_GA|gan|oc1861
p|MU_comp504097_c0_seq1|McaR|X
100
1
100
71
97
0.08

J

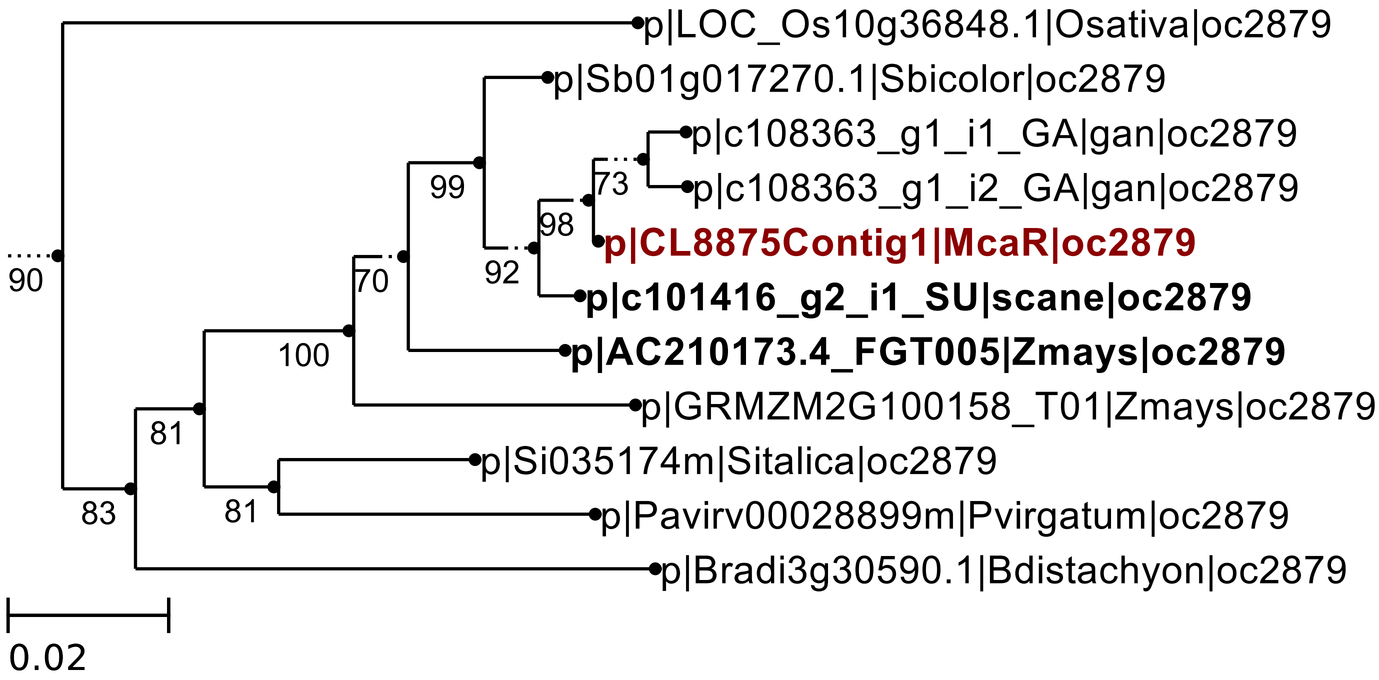
p|LOC_Os10g36848.1|Osativa|oc2879
p|Sb01g017270.1|Sbicolor|oc2879
p|c108363_g1_i1_GA|gan|oc2879
p|c108363_g1_i2_GA|gan|oc2879
p|CL8875Contig1|McaR|oc2879
p|c101416_g2_i1_SU|scane|oc2879
p|AC210173.4_FGT005|Zmays|oc2879
p|GRMZM2G100158_T01|Zmays|oc2879
p|Si035174m|Sitalica|oc2879
p|Pavirv00028899m|Pvirgatum|oc2879
p|Bradi3g30590.1|Bdistachyon|oc2879
99
73
98
92
70
90
100
81
83
81
0.02

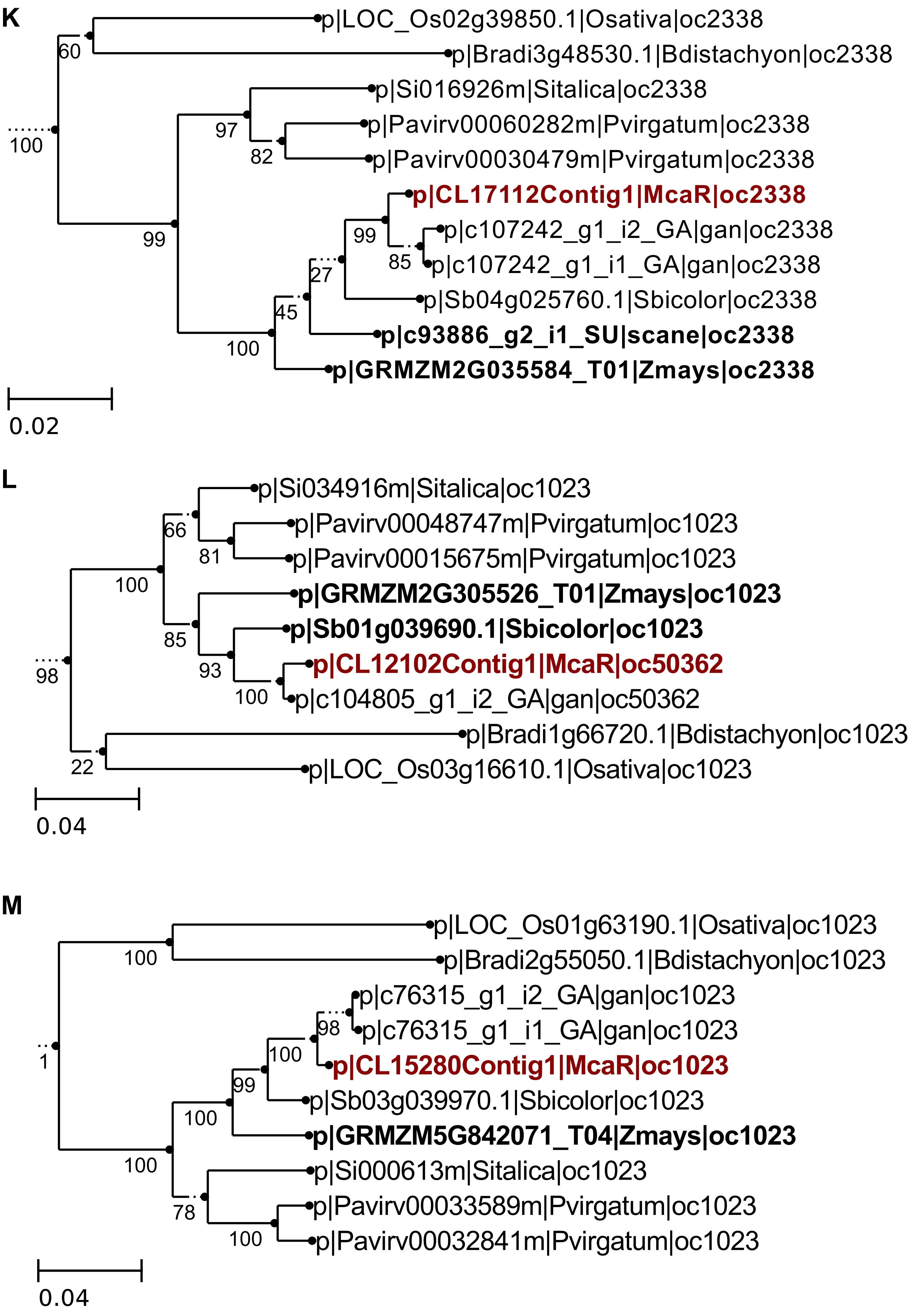
K
p|LOC_Os02g39850.1|Osativa|oc2338
p|Bradi3g48530.1|Bdistachyon|oc2338
p|Si016926m|Sitalica|oc2338
p|Pavirv00060282m|Pvirgatum|oc2338
p|Pavirv00030479m|Pvirgatum|oc2338
p|CL17112Contig1|McaR|oc2338
p|c107242_g1_i2_GA|gan|oc2338
p|c107242_g1_i1_GA|gan|oc2338
p|Sb04g025760.1|Sbicolor|oc2338
p|c93886_g2_i1_SU|scane|oc2338
p|GRMZM2G035584_T01|Zmays|oc2338
60
100
97
82
99
85
27
45
99
100
0.02
L
p|Si034916m|Sitalica|oc1023
p|Pavirv00048747m|Pvirgatum|oc1023
p|Pavirv00015675m|Pvirgatum|oc1023
p|GRMZM2G305526_T01|Zmays|oc1023
p|Sb01g039690.1|Sbicolor|oc1023
p|CL12102Contig1|McaR|oc50362
p|c104805_g1_i2_GA|gan|oc50362
p|Bradi1g66720.1|Bdistachyon|oc1023
p|LOC_Os03g16610.1|Osativa|oc1023
66
81
100
85
93
100
98
22
0.04
M
p|LOC_Os01g63190.1|Osativa|oc1023
p|Bradi2g55050.1|Bdistachyon|oc1023
p|c76315_g1_i2_GA|gan|oc1023
p|c76315_g1_i1_GA|gan|oc1023
p|CL15280Contig1|McaR|oc1023
p|Sb03g039970.1|Sbicolor|oc1023
p|GRMZM5G842071_T04|Zmays|oc1023
p|Si000613m|Sitalica|oc1023
p|Pavirv00033589m|Pvirgatum|oc1023
p|Pavirv00032841m|Pvirgatum|oc1023
100
98
100
99
1
100
100
78
100
0.04

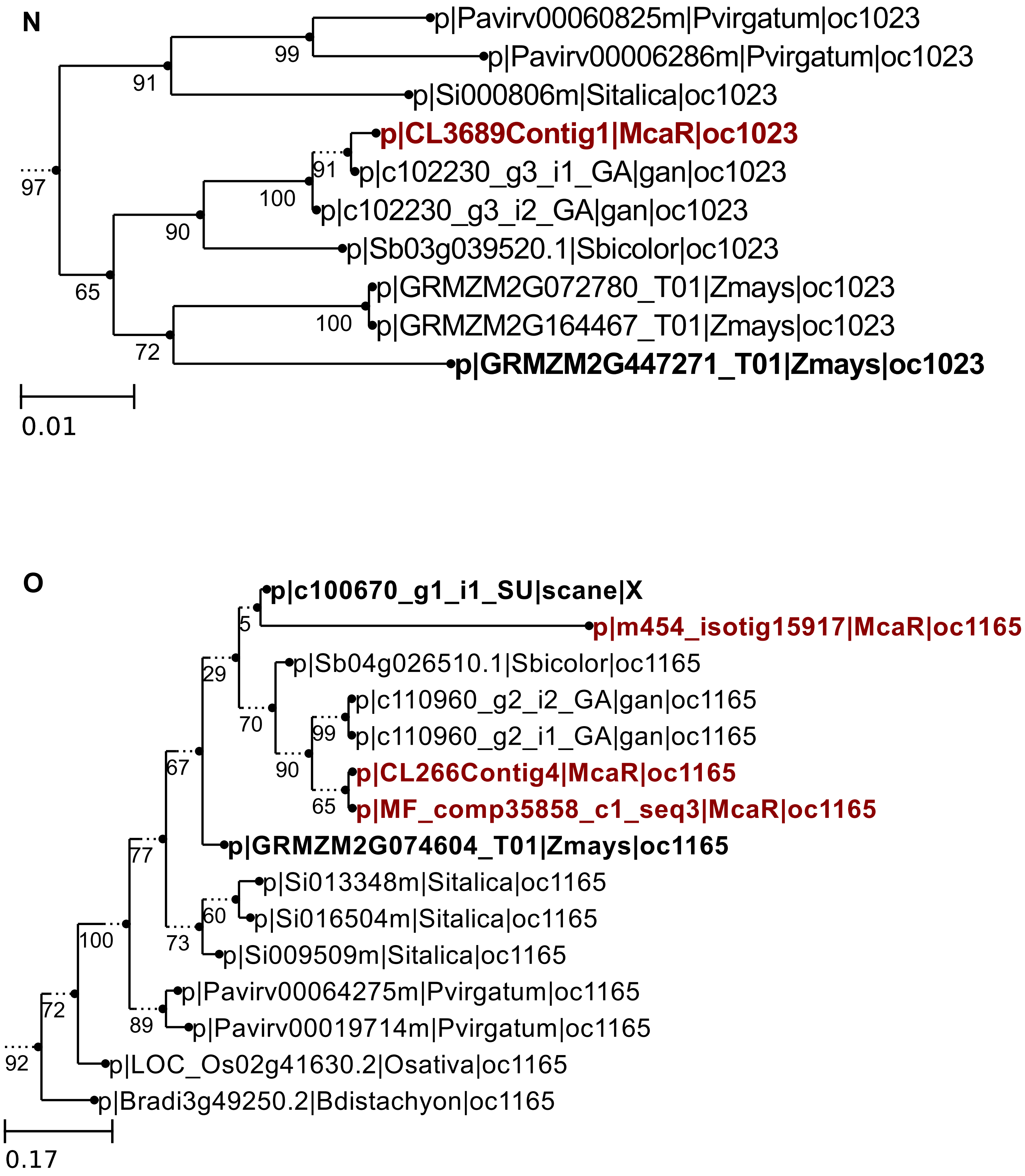
N
p|Pavirv00060825m|Pvirgatum|oc1023
p|Pavirv00006286m|Pvirgatum|oc1023
p|Si000806m|Sitalica|oc1023
p|CL3689Contig1|McaR|oc1023
p|c102230_g3_i1_GA|gan|oc1023
p|c102230_g3_i2_GA|gan|oc1023
p|Sb03g039520.1|Sbicolor|oc1023
p|GRMZM2G072780_T01|Zmays|oc1023
p|GRMZM2G164467_T01|Zmays|oc1023
p|GRMZM2G447271_T01|Zmays|oc1023
99
91
97
91
100
90
65
100
72
0.01
O
p|c100670_g1_i1_SU|scane|X
p|m454_isotig15917|McaR|oc1165
p|Sb04g026510.1|Sbicolor|oc1165
p|c110960_g2_i2_GA|gan|oc1165
p|c110960_g2_i1_GA|gan|oc1165
p|CL266Contig4|McaR|oc1165
p|MF_comp35858_c1_seq3|McaR|oc1165
p|GRMZM2G074604_T01|Zmays|oc1165
p|Si013348m|Sitalica|oc1165
p|Si016504m|Sitalica|oc1165
p|Si009509m|Sitalica|oc1165
p|Pavirv00064275m|Pvirgatum|oc1165
p|Pavirv00019714m|Pvirgatum|oc1165
p|LOC_Os02g41630.2|Osativa|oc1165
p|Bradi3g49250.2|Bdistachyon|oc1165
5
29
70
99
90
65
67
77
60
73
100
72
89
92
0.17

P

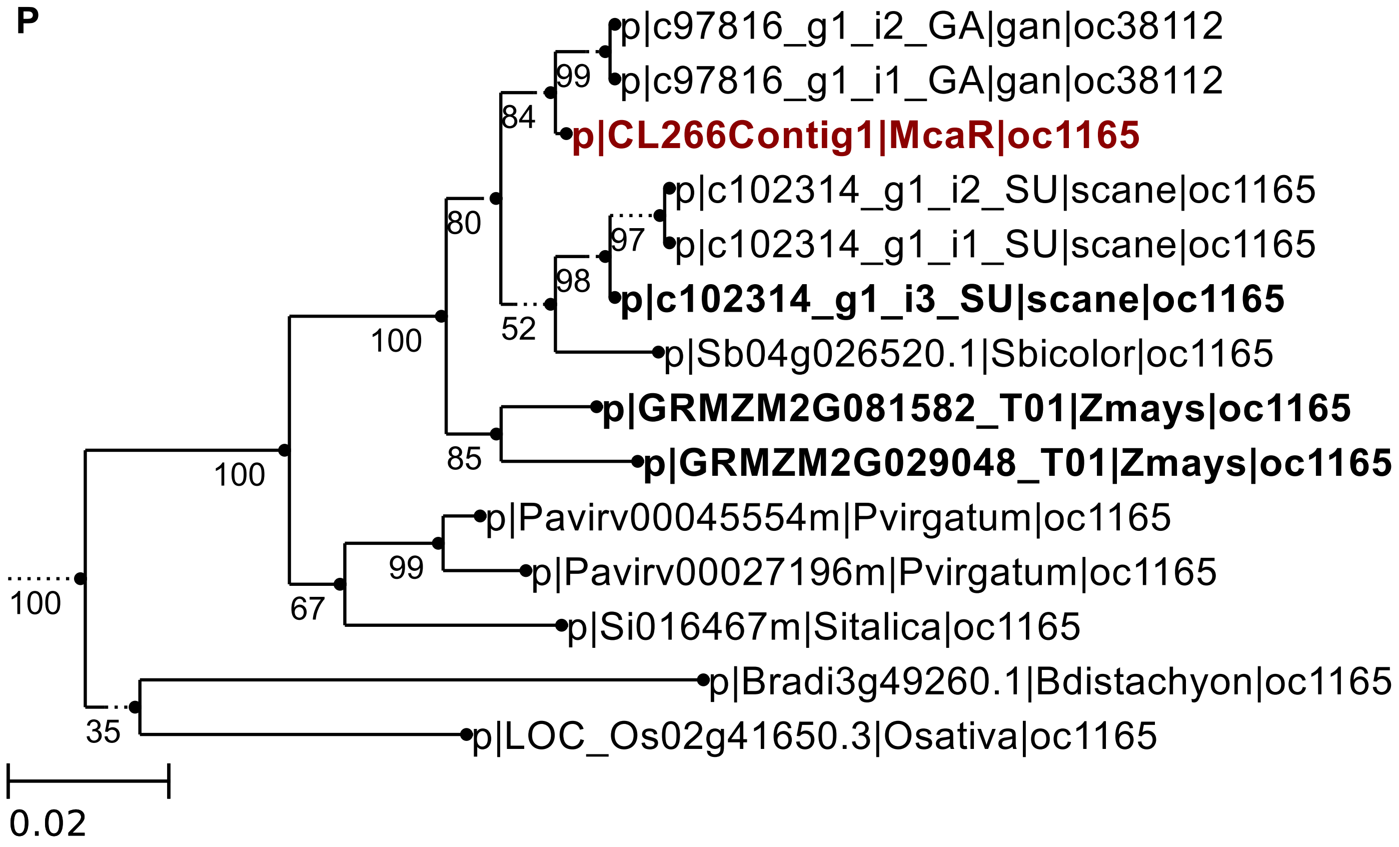
p|c97816_g1_i2_GA|gan|oc38112
p|c97816_g1_i1_GA|gan|oc38112
p|CL266Contig1|McaR|oc1165
p|c102314_g1_i2_SU|scane|oc1165
p|c102314_g1_i1_SU|scane|oc1165
p|c102314_g1_i3_SU|scane|oc1165
p|Sb04g026520.1|Sbicolor|oc1165
p|GRMZM2G081582_T01|Zmays|oc1165
p|GRMZM2G029048_T01|Zmays|oc1165
p|Pavirv00045554m|Pvirgatum|oc1165
p|Pavirv00027196m|Pvirgatum|oc1165
p|Si016467m|Sitalica|oc1165
p|Bradi3g49260.1|Bdistachyon|oc1165
p|LOC_Os02g41650.3|Osativa|oc1165
99
84
97
98
80
52
100
85
100
99
67
100
35
0.02
