# Supplementary material for: Orphan Crops Browser: a bridge between model and orphan crops
Source: Mol Breed. 2016 Jan 12;36:9. doi: 10.1007/s11032-015-0430-2 (PMC4710642; doi:10.1007/s11032-015-0430-2)
Supplement: Supplementary file 2 — Supplementary material 2 (PDF 100 kb) [file 11032_2015_430_MOESM2_ESM.pdf]

Supplemental Table S1. Lignin cell wall content of extremity sections of each harvested internode (upper and lower fragments), from the four *Miscanthus sinensis* genotypes. YI, young plant internode; MI, mature plant internode; low, lowest internode fragment; up, highest internode fragment.

	low	*stdev*	*up*	*stdev*
H0116-YI 2	14.62	0.33	16.34	0.27
H0116-YI 3	14.33	0.18	15.68	0.41
H0116-YI 4	13.07	0.15	15.60	0.14
H0117-YI 2	16.73	0.31	18.09	0.40
H0117-YI 3	15.97	1.07	17.11	0.21
H0117-YI 4	14.58	0.33	18.31	0.28
H0119-YI 2	17.88	0.08	18.47	0.30
H0119-YI 3	16.83	0.16	18.14	0.24
H0119-YI 4	16.27	0.28	17.48	0.41
H0120-YI 2	16.00	0.30	15.41	0.71
H0120-YI 3	14.27	0.04	16.33	0.26
H0120-YI 4	12.32	0.10	15.22	0.32

	low	*stdev*	*up*	*stdev*
H0116-MI 2	17.42	0.41	18.19	0.37
H0116-MI 3	16.93	0.13	18.08	0.46
H0116-MI 4	16.53	0.13	15.78	0.31
H0117- MI 2	18.66	1.16	19.27	0.56
H0117- MI 3	16.25	0.72	18.32	0.88
H0117- MI 4	17.90	0.25	17.51	0.49
H0119-MI 2	18.61	0.23	19.66	0.57
H0119-MI 3	19.26	0.16	19.02	0.39
H0119-MI 4	16.35	0.46	19.28	0.75
H0120-MI 2	17.94	0.36	18.10	0.20
H0120-MI 3	16.99	0.26	17.71	0.12
H0120-MI 4	15.80	0.29	16.98	0.31
