# Supplementary material for: Orphan Crops Browser: a bridge between model and orphan crops
Source: Mol Breed. 2016 Jan 12;36:9. doi: 10.1007/s11032-015-0430-2 (PMC4710642; doi:10.1007/s11032-015-0430-2)
Supplement: Supplementary file 3 — Supplementary material 3 (PDF 16 kb) [file 11032_2015_430_MOESM3_ESM.pdf]

Supplemental Table S2. List of primers used in the study. In red, adapter *attB* used for cloning and removed for quantification analyses.

fragment	fragment size	forward primer	reverse primer
Actin 1	147	CACCAGTTTCTGGGGAAGTTCGAC	GGTAACGAATCGGAGTCACTG
Ms4CL2	142	CACCTTGTTGCAAAGGAGGTCGTG	GTGTTGTCACTGCTCGGAAT
MsC3H1	146	CACCCAGCACTTTGTGGATGCACT	CTAACCAGCTCTGCCATTGC
MsC4H1a	141	CACCACGTGAAGAGGAACGCCA	TGAGCTTGTTGAAGAGCGGA
MsC4H1b	155	CACCGCTTCGAGTACAACTACGGC	CTCACCAGTCTGAGCCATCA
MsCAD2	133	CACCAGGCCAACGTTGAGCAGTA	GCCGGGATCTTCACCAAAAA
MsCCoAOMT1	203	CACCATCGCAGACGAGAAGAACCA	AAGTCACGGTAGAAGCGGAT
MsCCR1	220	CACCTGTCAGGAACCCAGATGACC	CGCGTTGATCACGTACTCC
MsCOMT1	148	CACCCCACGTCGACATGATCATGC	CTTGATGAACTCGATGGCCC
MsCOMTa	140	CACCATTAGCGGATCCTTGGTCGA	TTCACCTCGGTGTTAGTCGG
MsCOMTb	132	CACCAAGCTGTACCTCCCAGGGAT	GACGCCGTTGACAAACATGA
MsF5H1	136	CACCCTCCAAGACCCTCTGAGCTG	GGTCCATCATCGCCATGTTG
MsHCT1	134	CACCTTCTCCGGCCTGCATTTCAT	TACTCGATGTGCGGGTAGAC
MsLAC1	140	CACCGTGGTGGCGTTGGAGTACAA	GACGAGTCGTAGTTGCCGAA
MsLAC2	100	CACCGAAGCTCAGCTACAACTCGG	GAAGAAGTTGAAGCCGTGGA
MsLAC3	154	CACCACACGGAGGTGGTGATCAAT	AGCATGTACGTCTTCCCTGG
MsPAL1	134	CACCAGCTCATGTCGTCCACCTAC	AGAGGGGTTCATGGTCAGC
MsPAL2	123	CACCATGACGTCCACGTTCCTGAT	GTTCAGGCTCAGGGTCTTCT
