# Supplementary material for: Orphan Crops Browser: a bridge between model and orphan crops
Source: Mol Breed. 2016 Jan 12;36:9. doi: 10.1007/s11032-015-0430-2 (PMC4710642; doi:10.1007/s11032-015-0430-2)
Supplement: Supplementary file 4 — Supplementary material 4 (PDF 131 kb) [file 11032_2015_430_MOESM4_ESM.pdf]

Supplemental Table S3. Values from the quantitative expression analysis of the 17 lignin genes in three contrasting internode sections from four *M. sinensis* genotypes. Results are expressed as a mean of minimally three independent replicates, with each replicate containing at least two technical replicates.

			dCt			
Gene	Genotype	F-Probability	MIU2	YIL4	YIU2	LSD0.05
Ms4CL2	H0116	P<0.001	2.265	0.249	0.991	0.297
Ms4CL2	H0117	P=0.004	1.398	0.795	1.357	0.308
Ms4CL2	H0119	P<0.001	2.323	0.353	2.185	0.127
Ms4CL2	H0120	P<0.001	-0.141	-0.989	-0.555	0.069
MsC3H1	H0116	P<0.001	5.143	2.465	3.923	0.159
MsC3H1	H0117	P<0.001	3.755	2.133	3.707	0.149
MsC3H1	H0119	P<0.001	4.828	1.560	3.207	0.121
MsC3H1	H0120	P<0.001	1.997	-0.645	0.853	0.130
MsC4H1a	H0116	P<0.001	8.130	7.392	8.162	0.324
MsC4H1a	H0117	P<0.001	8.522	7.980	7.728	0.135
MsC4H1a	H0119	P=0.008	7.162	7.377	7.329	0.122
MsC4H1a	H0120	P<0.001	6.177	4.995	6.074	0.162
MsC4H1b	H0116	P<0.001	6.534	5.225	5.690	0.243
MsC4H1b	H0117	P<0.001	6.584	5.895	5.365	0.147
MsC4H1b	H0119	P<0.001	5.233	5.175	5.436	0.109
MsC4H1b	H0120	P<0.001	4.757	2.782	4.245	0.097
MsCAD2	H0116	P<0.001	5.394	3.740	4.440	0.302
MsCAD2	H0117	P<0.001	4.213	3.739	3.879	0.113
MsCAD2	H0119	P<0.001	4.095	3.648	4.590	0.086
MsCAD2	H0120	P<0.001	2.497	0.698	2.260	0.052
MsCCoAOMT1	H0116	P<0.001	1.958	-0.424	0.134	0.250
MsCCoAOMT1	H0117	P<0.001	1.785	0.736	0.855	0.131
MsCCoAOMT1	H0119	P<0.001	1.884	-0.211	1.118	0.127
MsCCoAOMT1	H0120	P<0.001	-0.411	-2.612	-1.329	0.087
MsCCR1	H0116	P<0.001	9.437	7.596	8.483	0.431
MsCCR1	H0117	P<0.001	8.818	8.126	8.159	0.136
MsCCR1	H0119	P<0.001	10.657	9.162	10.804	0.132
MsCCR1	H0120	P<0.001	6.077	4.260	5.927	0.106
MsCOMT1	H0116	P<0.001	0.500	-2.377	-0.967	0.073
MsCOMT1	H0117	P<0.001	-1.466	-2.149	-1.246	0.120
MsCOMT1	H0119	P<0.001	0.786	-2.603	-0.430	0.169
MsCOMT1	H0120	P<0.001	-2.696	-4.617	-3.707	0.122
MsCOMTa	H0116	P<0.001	0.691	1.025	0.097	0.232
MsCOMTa	H0117	P<0.001	1.356	2.442	0.778	0.577
MsCOMTa	H0119	P<0.001	0.301	0.562	-0.923	0.074
MsCOMTa	H0120	P<0.001	-0.259	-0.418	-1.061	0.095
MsCOMTb	H0116	P=0.007	2.219	3.460	2.104	0.759
MsCOMTb	H0117	P<0.001	3.938	6.646	4.751	0.927
MsCOMTb	H0119	P=0.002	3.954	5.017	2.029	1.174

MsCOMTb	H0120	P<0.001	3.770	4.927	2.322	0.157
MsF5H1	H0116	P<0.001	7.708	6.555	8.255	0.198
MsF5H1	H0117	P=0.006	6.347	6.180	6.857	0.370
MsF5H1	H0119	P<0.001	7.183	5.485	7.818	0.124
MsF5H1	H0120	P<0.001	4.112	1.878	4.405	0.121
MsHCT1	H0116	P<0.001	7.710	5.159	6.573	0.655
MsHCT1	H0117	P=0.006	6.286	5.991	6.493	0.269
MsHCT1	H0119	P<0.001	8.021	5.719	8.354	0.150
MsHCT1	H0120	P<0.001	5.219	3.684	5.153	0.117
MsLAC1	H0116	P<0.001	5.365	4.127	3.968	0.302
MsLAC1	H0117	P=0.013	2.240	2.303	1.955	0.223
MsLAC1	H0119	P<0.001	4.320	1.888	2.630	0.187
MsLAC1	H0120	P<0.001	0.778	-0.571	-0.034	0.124
MsLACa	H0116	P<0.001	9.625	9.120	9.948	0.338
MsLACa	H0117	P<0.001	9.950	9.238	9.333	0.270
MsLACa	H0119	P<0.001	8.112	7.778	8.990	0.160
MsLACa	H0120	P<0.001	7.767	8.038	8.470	0.250
MsLACb	H0116	P<0.001	6.028	2.582	4.464	0.770
MsLACb	H0117	P<0.001	3.169	2.210	2.920	0.246
MsLACb	H0119	P<0.001	5.373	2.217	5.122	0.093
MsLACb	H0120	P<0.001	1.503	-0.584	0.902	0.099
MsPAL1	H0116	P<0.001	3.137	-0.360	1.338	0.471
MsPAL1	H0117	P<0.001	1.689	0.401	1.121	0.410
MsPAL1	H0119	P<0.001	2.132	-0.281	1.869	0.114
MsPAL1	H0120	P<0.001	0.316	-1.730	0.048	0.100
MsPAL2	H0116	P<0.001	2.165	0.966	1.973	0.426
MsPAL2	H0117	P=0.258	1.767	1.672	1.738	0.124
MsPAL2	H0119	P<0.001	2.473	1.686	3.117	0.064
MsPAL2	H0120	P<0.001	0.533	-0.649	0.758	0.104
