# Supplementary material for: Orphan Crops Browser: a bridge between model and orphan crops
Source: Mol Breed. 2016 Jan 12;36:9. doi: 10.1007/s11032-015-0430-2 (PMC4710642; doi:10.1007/s11032-015-0430-2)
Supplement: Supplementary file 5 — Supplementary material 5 (PDF 99 kb) [file 11032_2015_430_MOESM5_ESM.pdf]

Supplemental Table S4. Phylogenetic tree construction details

Miscanthus gene ID	*Supplemental Figure*	*Details*
Ms4CL2	S1-A	The best blastn hit of SCVPLB1015F12.g is c88946_g1_i2_SU from cluster oc26599. This cluster is small (4 entries) and only contains sequences from miscanthus, sugarcane and *Vitis venifera*. A seed blast (max hits 100) was performed in order to retrieve additional sequences. A codon sequence alignment was created from the retrieved sequences and cropped to the region of the alignment containing sequence c88946_g1_i2_SU. The alignment was further manually trimmed in order to remove lowly conserved regions.
MsC3H1	S1-B	A blastn search reveals that SCVPCL6041E07.g corresponds to c70890_g1_i1_SU. This sequence, which is relatively short, does not belong to any cluster. A seed blast was performed in order to retrieve extra sequences for the construction of a codon alignment. The alignment was cropped according to c70890_g1_i1_SU and manually trimmed.
MsC4Ha and *b*	S1-C	The best hit for SCCCCL4009H01.g, c94596_g1_i1_SU, belongs to cluster oc1757. The codon alignment constructed from the cluster members was cropped according to c94596_g1_i1_SU and afterward trimmed. The trimming was performed in such matter that no *Miscanthus sinensis* and sugarcane sequences had long terminal gaps in the alignment.
MsCAD2	S1-D	SCBFAM2021E08.g and SCEPRZ1011A02.g have similar sets of blastn hits containing c91614_g1_i2_SU, c91614_g1_i1_SU and c91614_g1_i3_SU. Whereas c91614_g1_i3_SU belongs to a small cluster, c91614_g1_i2_SU and c91614_g1_i1_SU belong to a larger cluster oc4288. The codon alignment created from cluster oc4288 members was cropped to the c91614_g1_i2_SU and c91614_g1_i1_SU and terminal gaps were manually removed.
MsCCoAOMT1	S1-E	Visual inspection of the blastn results indicates that c107456_g2_i1_SU is the best hit of all four ESTs. The alignments for SCCCLR1079A02 are particularly good. c107456_g2_i1_SU belongs to cluster oc5509. Although, it was expected based upon its annotation, GRMZM2G099363 was a member of this cluster. Therefore a seed blast (max hits 100 and minimum coverage 60%) was performed which did retrieve the maize protein. The codon alignment of seed blast results was cropped to c107456_g2_i1_SU and lowly conserved regions were removed manually.
MsCCR1	S1-F	GRMZM2G131205 belongs to cluster oc2454. The codon alignment of the cluster member was cropped to GRMZM2G131205 and then manually edited to remove lowly conserved regions.
MsCOMT1	S1-G	The best blast hits of SCRFLR1012F12.g are c96304_g1_i2_SU and c96304_g1_i1_SU, which both belong to cluster oc4123. The codon alignment of the cluster was cropped to both sugarcane proteins and then trimmed in such matter that no miscanthus sequence had long terminal gaps.
MsCOMTa	S1-H	GRMZM2G141026 belongs to cluster oc1861. The codon alignment of the cluster was trimmed to GRMZM2G141026. Lowly conserved regions were removed and it was ensured that no miscanthus sequence had long terminal gaps. GSVIVT01020647001 was removed from the alignment, as it was the only sequence with a long terminal gap after trimming.
MsCOMTb	S1-I	GRMZM2G140996 was also present in the tree constructed for *MsCOMTa*. Although, no *Miscanthus sinensis* protein was clustered together with the maize protein, there was a *Miscanthus x. giganteus* protein. The cDNA sequence corresponding to the *M. x. giganteus* protein with id c97140_g1_i3_GA was searched against

		the *M. sinensis* sequences in our database. The best hit (98% identity) was MU_comp504097_c0_seq1. A seed blast was performed with this miscanthus sequence and a codon alignment was generated from the results. The alignment was cropped to the miscanthus sequence and manually trimmed to remove lowly conserved regions.
MsF5H1	S1-J	The best blast hit of SCJLRT1022E04.g is c101416_g2_i1_SU, which is a member of cluster oc2879. The codon alignment of the cluster was cropped to c101416_g2_i1_SU. The alignment was trimmed in such way that no miscanthus sequences had long terminal gaps. Lowly conserved regions in the alignment were removed manually.
MsHCT1	S1-K	The best of SCCCCL4009E02.g was not against sugarcane. Through visual inspection it was considered that the best hit against sugarcane was c93886_g2_i1_SU. The score of this alignment was merely 3 bits lower than the best sugarcane hit reported by blast. c93886_g2_i1_SU belongs to cluster oc2338. The codon alignment created from the cluster members was cropped to c93886_g2_i1_SU and manually trimmed. After trimming, no sugarcane and miscanthus had long terminal gaps.
MsLAC1	S1-L	SCUTST3084C11.g did not have a sugarcane hit in the database. Its best hit was a sorghum protein with id Sb01g039690.1. A seed blast was performed with the sorghum sequence. A codon alignment was constructed and cropped to the sorghum protein and manually trimmed. No sugarcane or miscanthus sequence had long terminal gaps after trimming.
MsLACa	S1-M	For GRMZM5G842071 a seed blast was performed (40% coverage). The codon alignment of the results was cropped to GRMZM5G842071. Lowly conserved regions were manually removed and no miscanthus sequence had long terminal gaps.
MsLACb	S1-N	The phylogeny for the GRMZM2G447271 mays gene was resolved in the tree constructed for *MsLAC1*.
MsPAL1	S1-O	The best hit for SCCCLR1048D07.g is c100670_g1_i1_SU, which does not belong to any cluster. SCJFLR1017B11.g did not have very good hits against the database. A seed blast was performed with c100670_g1_i1_SU and a codon alignment was generated. The alignment was cropped to c100670_g1_i1_SU and manually trimmed. After trimming no miscanthus sequences had long terminal gaps.
MsPAL2	S1-P	The best hits of SCEQRT1024E12.g are c102314_g1_i3_SU, c102314_g1_i1_SU and c102314_g1_i2_SU. The phylogenetic tree for these sequences was already solved in the procedure for *MsPAL1*.
